# Effects of Oral Cannabinoids on Systemic Inflammation and Viral Reservoir Markers in People with HIV on Antiretroviral Therapy: Results of the CTN PT028 Pilot Clinical Trial

**DOI:** 10.3390/cells12141811

**Published:** 2023-07-08

**Authors:** Ralph-Sydney Mboumba Bouassa, Eve Comeau, Yulia Alexandrova, Amélie Pagliuzza, Alexis Yero, Suzanne Samarani, Judy Needham, Joel Singer, Terry Lee, Florian Bobeuf, Claude Vertzagias, Giada Sebastiani, Shari Margolese, Enrico Mandarino, Marina B. Klein, Bertrand Lebouché, Jean-Pierre Routy, Nicolas Chomont, Cecilia T. Costiniuk, Mohammad-Ali Jenabian

**Affiliations:** 1 Department of Biological Sciences and CERMO-FC Research Centre, Université du Québec à Montréal, Montreal, QC H2X 3Y7, Canada; 2 Infectious Diseases and Immunity in Global Health Program, Research Institute of the McGill University Health Centre, Montreal, QC H4A 3J1, Canada; suzanne.samarani@muhc.mcgill.ca (S.S.); giada.sebastiani@mcgill.ca (G.S.);; 3 Centre de Recherche du Centre Hospitalier de l’Université de Montréal, Montreal, QC H2X 0A9, Canada; amelie.pagliuzza.chum@ssss.gouv.qc.ca (A.P.); nicolas.chomont@umontreal.ca (N.C.); 4 Department of Medicine, Division of Infectious Diseases and Chronic Viral Illness Service, McGill University Health Centre, Montreal, QC H4A 3J1, Canada; 5 CIHR Canadian HIV Trials Network, Vancouver, BC V6Z 1Y6, Canada; 6 Centre for Health Evaluation and Outcome Sciences, St. Paul’s Hospital, Vancouver, BC V6Z 1Y6, Canada; 7 School of Population and Public Health, University of British Columbia, Vancouver, BC V6T 1Z4, Canada; 8 Department of Family Medicine, Faculty of Medicine and Health Sciences, McGill University, Montreal, QC H4A 3J1, Canada; 9 Centre for Outcomes Research & Evaluation, Research Institute of the McGill University Health Centre, Montreal, QC H4A 3J1, Canada; 10 Département de Microbiologie, Infectiologie et Immunologie, Université de Montréal, Montreal, QC H3T 1J4, Canada

**Keywords:** cannabinoids, Δ9-tetrahydrocannabinol (THC), cannabidiol (CBD), systemic inflammation, immune response, HIV reservoir

## Abstract

Chronic HIV infection is characterized by persistent inflammation despite antiretroviral therapy (ART). Cannabinoids may help reduce systemic inflammation in people with HIV (PWH). To assess the effects of oral cannabinoids during HIV, ten PWH on ART were randomized (*n* = 5/group) to increasing doses of oral Δ9-tetrahydrocannabinol (THC): cannabidiol (CBD) combination (2.5:2.5–15:15 mg/day) capsules or CBD-only (200–800 mg/day) capsules for 12 weeks. Blood specimens were collected prospectively 7–21 days prior to treatment initiation and at weeks 0 to 14. Plasma cytokine levels were determined via Luminex and ELISA. Immune cell subsets were characterized by flow cytometry. HIV DNA/RNA were measured in circulating CD4 T-cells and sperm by ultra-sensitive qPCR. Results from both arms were combined for statistical analysis. Plasma levels of IFN-γ, IL-1β, sTNFRII, and REG-3α were significantly reduced at the end of treatment (*p* ˂ 0.05). A significant decrease in frequencies of PD1+ memory CD4 T-cells, CD73+ regulatory CD4 T-cells, and M-DC8+ intermediate monocytes was also observed (*p* ˂ 0.05), along with a transient decrease in CD28–CD57+ senescent CD4 and CD8 T-cells. Ki-67+ CD4 T-cells, CCR2+ non-classical monocytes, and myeloid dendritic cells increased over time (*p* ˂ 0.05). There were no significant changes in other inflammatory markers or HIV DNA/RNA levels. These findings can guide future large clinical trials investigating cannabinoid anti-inflammatory properties.

## 1. Introduction

Despite effective antiretroviral therapy (ART), people with HIV (PWH) continue to suffer from chronic systemic inflammation and persistent immune activation [1,2,3]. This deleterious inflammatory state is thought to result from multifactorial and progressive events, including CD4 T-cell depletion in gut-associated lymphoid tissue (GALT) during acute infection, which induces persistent dysregulation of intestinal T-cell homeostasis and promotes long-lasting disruption of the gut epithelial mucosa [1,4]. Increased gut mucosal permeability leads to microbial antigen translocation from the gut lumen into the bloodstream [1,2,3,5,6]. These antigens, in turn, trigger immune cell activation and subsequent release of high amounts of pro-inflammatory soluble factors. Gut mucosal damage in PWH is chronic, hence it persists in PWH despite HIV infection being controlled with ART and regardless of the timing of ART initiation [7,8]. It leads to persistent T-cell activation and their subsequent exhaustion and immunosenescence [1,2,3,5,9]. Together, these factors contribute to the establishment of a vicious cycle that fuels a chronic inflammatory state, leading to early ageing and predisposing PWH to an increased risk of non-AIDS co-morbidities such as metabolic syndrome, cardiovascular diseases, cancers, and neurological disorders [1,2,3,5,6]. In addition, heightened levels of inflammation during ART are associated with the persistence of HIV reservoirs [10,11], the major obstacle to HIV eradication. Thus, targeting various players involved in this vicious cycle could help dampen chronic inflammation, reduce the occurrence rate of non-AIDS comorbidities in PWH, and facilitate the clearance of HIV reservoirs.

Primary phytocannabinoids ∆9-tetrahydrocannabinol (THC) and cannabidiol (CBD) displayed anti-inflammatory properties in experimental models in both in vitro [12,13,14,15,16] and in vivo studies involving mice [17], simian immunodeficiency virus (SIV) infection of non-human primates [18,19,20,21] and in humans [22]. Cannabinoids improved experimental inflammation by suppressing the release of pro-inflammatory cytokines and reactive oxygen species [23], reducing frequencies of inflammatory T helper (Th) 17 lymphocytes, and increasing frequencies of regulatory T-cells (Tregs) [24,25]. Cannabinoids also attenuated SIV-associated intestinal inflammation [18], as well as SIV-induced neuroinflammation, by reducing gut microbiome dysbiosis [26]. The latter occurred via increased gut bacterial diversity, decreased expression of pro-inflammatory genes, and increased production of anti-inflammatory regulatory micro-RNA in the gastrointestinal tract [20,26,27,28]. THC and CBD exert their anti-inflammatory properties largely through the activation of receptors of the endocannabinoid system, particularly the cannabinoid receptor type 1 (CB1R) and 2 (CB2R), which are mainly expressed, although at different levels, in the brain and the central nervous system (CNS) [29,30], the gastrointestinal tract [25,31,32] and on immune cells [33]. By alleviating gut epithelial damage and systemic inflammation, cannabinoid treatment could have the potential to reduce systemic inflammation in PWH [34,35]. Furthermore, oral administration of lipid-based formulations of cannabinoids could substantially facilitate the exposure of intestinal tissue-resident immune cells to these molecules and promote their anti-inflammatory effects [36].

Recreational cannabis was legalized in Canada in 2018, making it possible to possess cannabis without the need for a prescription. Cannabis use is common among PWH for both recreational and medicinal purposes. Many PWH use it to alleviate various symptomatologies such as anxiety, depression, and chronic pain [37,38,39,40]. Similarly to purified cannabinoids, consumption of the cannabis plant via inhalation has also been associated with anti-inflammatory outcomes in human observational studies [32,41,42,43,44,45]. However, because the cannabis plant contains more than 120 bioactive compounds, including phytocannabinoids [46], disentangling the effects of specific compounds, their doses, and their observed effects is challenging if not impossible. In addition, one of the main phytocannabinoids, THC, is well known for its psychotropic effects, which has been associated with adverse effects [46]. Cannabis consumption has also been associated with some negative outcomes, such as decreased adherence to ART in older PWH [47,48]. In adolescents, cannabis use has been associated with impaired cognitive abilities, an observation which has been attributable to CB1R activation in the CNS and the brain by THC [49].

Given the discrepancies across studies and the inherent risk of confounding in observational studies, it is important to clearly delineate whether cannabinoids may have a role in reducing systemic inflammation in PWH. In addition, it is necessary to determine whether cannabinoids, when administered at safe and tolerable doses to PWH, can have an impact on markers of systemic inflammation. We recently reported that cannabinoids administered in a randomized, open-label, interventional clinical trial (CTN PT028 study) were generally safe and well tolerated in PWH with well-controlled HIV on ART [50]. Herein, we report on the effects of oral cannabinoids on systemic inflammation, markers of gut mucosal damage, lymphoid and myeloid immune cell subsets, and markers of HIV persistence in PWH under suppressive ART. Given their synergistic effect, and the fact that CBD tends to improve the tolerability of THC when administered together [51,52,53,54], a THC:CBD combination was selected for one arm of this study. A formulation containing only CBD was also selected, in order to document effects related to the use of CBD in isolation.

## 2. Materials and Methods

### 2.1. Study Population and Design

This study was part of a randomized, open-label, interventional pilot clinical trial (CIHR Canadian HIV Trials Network (CTN) PT028, Trial registration number: NCT03550352) aiming to assess the safety and tolerability of oral THC:CBD combined, or CBD-only capsules consumed daily for 12 weeks with an initial target sample size of *n* = 26 (*n* = 13 per study arm) [50]. Participants were enrolled between September 2021 and February 2022. PWH (18 years and older) on ART for at least 3 years with suppressed viral loads (VL < 40 copies/mL) were recruited at the Chronic Viral Illness Service, Royal Victoria Hospital of the McGill University Health Centre in Montreal, Canada. Participants were randomized in a 1:1 ratio and received oral capsules of highly purified (>98%) cannabinoid oil (Tilray Brands, Inc., New York City, NY, USA), consisting of either TN-TC11M2 formulation, a THC:CBD combination in a 1:1 ratio (CBD: 2.5/THC: 2.5 mg), or TN-C200M2 formulation, containing CBD only (200 mg), for a maximum of 12 weeks. The following up-titration schedule was recommended to participants in THC:CBD study arm: 5 mg THC/5 mg CBD (1 capsule twice daily) during week 0 and week 1; 10 mg THC/10 mg CBD (2 capsules twice daily) week 2 and week 3; and 15 mg THC/15 mg CBD (2 capsules three times daily) week 4 to week 12 (end of treatment). For the CBD-only study arm, participants were advised to take 200 mg CBD (1 capsule once daily) from week 0 to week 1; 400 mg CBD (1 capsule twice daily) from week 2 to week 3; and 800 mg CBD (2 capsules twice daily) from week 4 to week 12. The dosages for the THC/CBD combination (2.5–15 mg/day) and the CBD-only formulation (200–800 mg/day) have been determined based on other clinical trials that have shown their safety, tolerability, and efficacy for the management of chronic pain, epilepsy, schizophrenia or multiple sclerosis [55,56,57,58]. The decision to use these specific formulations was also influenced in part by availability from the supplier. Of note, we chose a formulation of oral capsules for this study in order to have accurate information regarding the doses of cannabinoids participants were ingesting, to avoid the pulmonary toxicity associated with inhalation, and to determine whether oral administration can have an impact on markers associated with gut mucosal translocation. Participants were excluded if they used cannabinoid-containing products outside of this study or within 4 weeks before starting this study. Cannabinoids were permanently discontinued when severe adverse events occurred [50]. Further details on study design, participant recruitment, and inclusion/exclusion criteria can be found in the study protocol [59] and our previous publication on this trial which reported on safety and tolerability [50]. 

### 2.2. Blood and Semen Specimens Processing

Blood specimens were collected from each participant 7 to 21 days prior to treatment initiation, at week 0, and weeks 1, 2, 6, 8, 12, and 14 (end of study) for hematological/biochemical profile assessment [50], plasma cytokine and gut damage measurements, characterization of immune cell subsets, and quantification of HIV reservoir markers. Blood plasma was isolated via centrifugation, and stored at −80 °C to be analyzed in one batch at the end of this study. Peripheral blood mononuclear cells (PBMCs) were isolated using lymphocyte separation medium (WISENT Inc., Quebec City, QC, Canada) and cryopreserved in fetal bovine serum (FBS; WISENT Inc., Quebec City, QC, Canada) containing 10% dimethyl sulfoxide (DMSO) to be analyzed after study completion.

In parallel, semen samples were collected in sterile containers with 10 mL of Roswell Park Memorial Institute medium (RPMI; WISENT Inc., Quebec City, QC, Canada) with 100 U/mL penicillin and 100 mg/mL streptomycin (WISENT Inc., Quebec City, QC, Canada) were obtained from male participants (21 to 7 days prior to treatment initiation and at treatment completion at week 12). Upon arrival at the clinic, semen specimens were sent to the lab and centrifuged. The supernatant and cell pellets were separated and stored at −80 °C until HIV reservoir quantification. 

### 2.3. Measurements of Soluble Markers and Cytokines in Plasma

Tumor necrosis factor alpha (TNF-α), interferon-gamma (IFN-γ), interleukin 1-beta (IL-1β), IL-6, IL-8, interferon gamma inducible protein-10 (IP-10) and IL-10 were measured in 25 μL of plasma using the MILLIPLEX^®^ Human Cytokine/Chemokine/Growth Factor Panel A according to the manufacturer’s instructions (MilliporeSigma, Burlington, MA, USA). ELISA kits were used to quantify lipopolysaccharide (LPS) (Cusabio Technology LLC, Houston, TX, USA), soluble CD14 (sCD14) (Hycult Biotech, Uden, Netherlands), soluble CD27 (sCD27) (Thermo Fisher Scientific, Waltham, MA, USA), soluble receptor for tumor necrosis factor type II (sTNFRII) (R&D Systems, Inc., Minneapolis, MN, USA), intestinal fatty acid binding protein (I-FABP) (Hycult Biotech, Uden, Netherlands), and human regenerating islet derived protein 3 alpha (REG-3α) (R&D Systems, Inc., Minneapolis, MN, USA) according to the manufacturer’s protocols. All measurements were performed in duplicate. 

### 2.4. Ex Vivo Immunophenotyping of T-Cells, Monocytes, and Dendritic Cells

Multiparametric flow cytometry was used for the immunophenotyping of T-cells, monocytes, and dendritic cells. 1 × 10^6^ PBMCs were stained with extracellular antibodies in phosphate-buffered saline (PBS) + 2% FBS for 1 h at 4 °C protected from light. For intracellular staining, cells were fixed and permeabilized using the Transcription Factor Buffer Set according to the manufacturer’s instructions (BD Bioscience, ON, Canada), and incubated with the appropriate antibodies for 1 h at 4 °C protected from light. Antibody-labeled cells were acquired on a 3-laser BD Fortessa-X20. Antibodies used for phenotyping are listed in Table S1, and fluorochrome minus one control (FMO) were used for some markers such as CCR6, CCR4, CXCR3, CX3CR1, CD163, and CCR2. All flow cytometric data were analyzed using FlowJo V10.8.1 (FlowJo LLC, Ashland, OR, USA). 

### 2.5. HIV DNA and Cell-Associated HIV RNA Quantification

Prior to quantification, genomic DNA and cellular RNA were extracted from PBMCs and semen cell pellets using the QIAamp DNA mini kit and QIAamp RNA mini kit (Qiagen, Hilden, Germany), respectively. Total HIV DNA and cell-associated HIV RNA targeting the LTR-gag region were measured in blood CD4 T-cells and sperm cells by ultra-sensitive nested real-time PCR, as previously described [60,61]. Cell-free viral RNA was also measured in semen supernatant by ultra-sensitive qPCR [60,61]. Detailed methodology is provided in Supplementary Material S1. 

### 2.6. Statistical Analyses

Descriptive statistics of quantitative variables were presented as the means with standard deviations and medians with interquartile range (IQR). The Wilcoxon matched-pairs signed-rank test was used to compare paired repeated measurements between two visits. Due to the small number of participants who have completed the full treatment course (*n* = 8), and because each participant underwent a personalized cannabinoid titration schedule according to their tolerability, study results from both arms were pooled together for non-parametric paired statistical analysis. The primary analysis has been performed between the initiation of the cannabinoid treatment (week 0) versus the treatment interruption (week 12) as well as two weeks after treatment interruption (week 14) to assess the persistence of cannabinoid effects. Secondly, a week-to-week comparison is also provided as a supplementary analysis. GraphPad Prism Software (version 9.0.0, San Diego, CA, USA) was used for statistical analyses. 

## 3. Results

### 3.1. Study Participants

Despite the initial sample size target of *n* = 26, this study was ended prematurely due to the rupture of cannabinoid capsules stock, the impossibility of renewing the stock of capsules with the same manufacturing criteria, and enrolment challenges as previously reported [50]. Thus, 10 PWH (median age: 57.5 years, IQR: 55–62), 8 males and 2 females, were included over a 6-month period, randomized in a 1:1 ratio to either TN-TC11M2 (arm 1) or TN-C200M2 (arm 2) [50]. Their baseline characteristics are summarized in Table 1. Eight study participants successfully completed the treatment and 2 were withdrawn for safety reasons as described previously [50]. CD4 T-cell count and CD4/CD8 ratio were stable and HIV viral load remained suppressed throughout the study [50]. All participants abstained from cannabis smoking and cannabis edibles for at least 4 weeks before study initiation and over time of the study duration. The majority (8/10) of participants reported at least one adverse event, and most of them were of mild to moderate severity. The frequently reported were somnolence (50%), diarrhea (20%), difficulty concentrating (20%), transaminitis (20%), and worsened diabetes type 2 (20%). A complete list of adverse event that have occurred during this study was previously reported [50]. Somnolence, difficulty concentrating, cognitive impairment, and increased appetite were considered definitively related to cannabinoids intake. 

### 3.2. Effect of Oral Cannabinoids on Plasma Markers of Gut Epithelial Damage, Microbial Translocation, and Systemic Inflammation

#### Reduced Levels of Soluble Markers of Gut Epithelial Damage, Microbial Translocation, Immune Activation, and Pro-Inflammatory Cytokines

**Figure 1:**
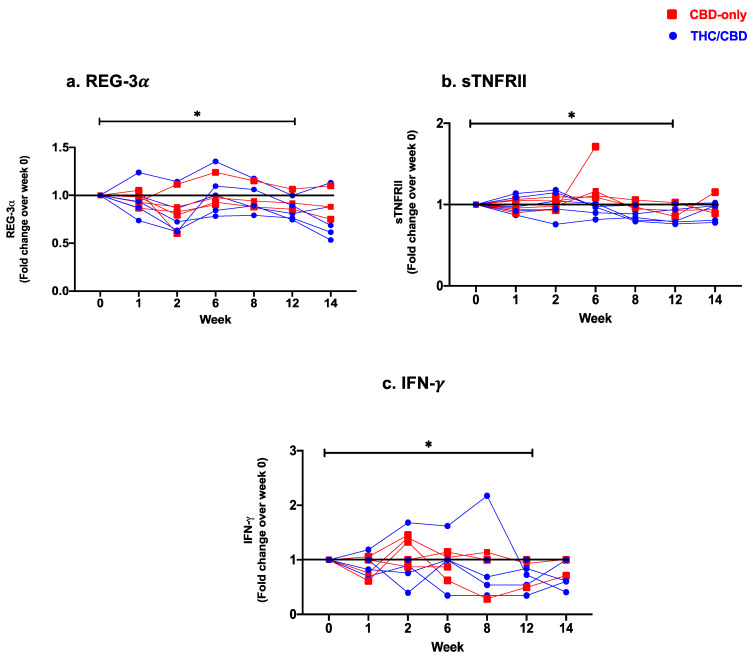
Fold change expression over 12 weeks versus weeks 0 of CBD-only (red square) and THC/CBD combination (blue circle), (**a**) Regenerating islet derived protein 3 alpha (REG-3α), (**b**) Soluble receptor for tumor necrosis factor type II (sTNFRII), and (**c**) Interferon-gamma (IFN-γ). * *p* < 0.05.

The effects of cannabinoid treatment on the integrity of the gut mucosal barrier were evaluated through changes in plasma levels of I-FABP and REG-3α as markers of gut epithelial damage in PWH [7,62]. The overall plasma levels of REG-3α were significantly lower after treatment completion (week 0 vs. week 12: *p* = 0.04, Figure 1a). Following treatment initiation, a significant decrease in levels of REG-3α was observed at week 2 (week 0 vs. week 2: *p* = 0.001), which increased transiently at week 6 (week 2 vs. week 6: *p* = 0.004), and then continued to drop until the end of treatment (week 6 vs. week 12: *p* = 0.008). No significant changes in levels of I-FABP were observed (Table 2). 

Plasma levels of bacterial LPS did not significantly change from baseline until treatment completion. However, plasma levels of LPS showed a transient increase at week 8 (week 0 vs. week 8: *p* = 0.008), followed by a significant drop up until treatment termination (week 8 vs. week 12: *p* = 0.02) (Table 2). Plasma markers of immune activation (sCD14, sCD27, sTNFRII) were also assessed. sCD14 is shed by activated monocytes and serves as a marker of monocyte activation and inflammation during HIV infection [7,62]. As with LPS, no significant changes in the plasma level of sCD14 were observed from baseline until treatment completion. sCD14 also followed a biphasic pattern with a slight increase after cannabinoid treatment initiation (week 1 vs. week 6 *p* = 0.02), followed by a significant drop in its plasma concentration until the end of treatment (week 6 vs. week 8: *p* = 0.02; week 6 vs. week 12: *p* = 0.02). sTNFRII is a soluble form of the TNF-α receptor and serves also as a marker of inflammation in HIV infection [63]. Levels of sTNFRII dropped significantly at week 8 (week 0 vs. week 8: *p* = 0.04) and remained low until the end of treatment (week 0 vs. week 12: *p* = 0.04, Figure 1b). Lastly, we have assessed plasma levels of sCD27, which is shed from the surface of activated lymphocytes, and serves as a marker of T-cell–mediated inflammation [64]. There were no significant changes in levels of sCD27 over the course of treatment (Table 2). Plasma levels of pro-inflammatory (TNF-α, IFN-γ, IL-1β, IL-6, IL-8, IP-10) and anti-inflammatory (IL-10) cytokines were measured by Luminex. A significant decrease in plasma levels of TNF-α (week 0 vs. week 14; *p* = 0.02), IFN-γ (week 0 vs. week 12: *p* = 0.03, Figure 1c), IL-1β (week 1 vs. week 12; *p* = 0.02), and IL-8 (week 8 vs. week 14; *p =* 0.03) was observed (Table 2). Other soluble markers showed a biphasic pattern with an early increase followed by a reduction until the end of the treatment. Plasma levels of IP-10 showed a transient drop at week 6 (week 1 vs. week 6: *p* = 0.01) and a subsequent rebound at week 8 (week 6 vs. week 8: *p* = 0.04), with no overall significant change between initiation and end of treatment. No significant changes in levels of IL-6 or IL-10 were observed (Table 2). 

Altogether, over the treatment period, the dynamic of most of the soluble plasma markers showed a biphasic pattern, with a transient increase during the first weeks of cannabinoids uptake, followed by a significant reduction until treatment completion.

### 3.3. Effect of Oral Cannabinoids on Blood T-Cell, Monocyte, and Dendritic Cell Subsets

#### 3.3.1. Changes in Circulating CD4 T-Cell Subsets

While no significant changes in frequencies of naïve (CD45RA+CD28+CCR7+), central memory (CD45RA−CD28+CCR7+), and transitional memory (CD45RA−CD28+CCR7−) CD4 T-cells were observed, levels of effector memory (CD45RA−CD28−CCR7−) CD4 T-cells were significantly lower at weeks 6 and 8 following treatment initiation (week 0 vs. week 6; *p* = 0.04, week 1 vs. week 8; *p* = 0.02) (Table 3, Figure S1). Similarly, frequencies of terminally differentiated (CD45RA+CD28−CCR7−) CD4 T-cells were significantly lower at week 8 (week 1 vs. week 8: *p* = 0.02). However, in both cases, the overall change in these memory subsets between week 0 and week 12 was not significant. 

**Figure 2:**
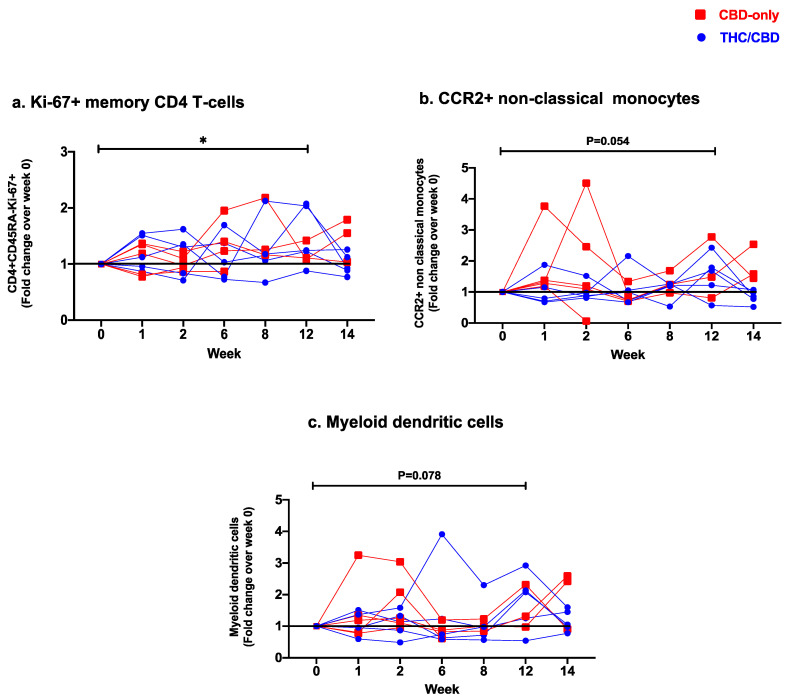
Fold change expression over 12 weeks versus weeks 0 of CBD-only (red square) and THC/CBD combination (blue circle), (**a**) Ki-67+ memory CD4 T-cells, (**b**) CCR2+ non-classical monocytes, and (**c**) Myeloid dendritic cells. * *p* < 0.05.

Frequencies of senescent (CD28−CD57+) CD4 T-cells dropped significantly between weeks 1 and 8 (*p =* 0.04). Levels of PD1+ CD45RA−CD4 T-cells also decreased significantly over the treatment course (week 0 vs. week 14: *p* = 0.02, week 1 vs. week 12: *p* = 0.02, week 1 vs. week 14: *p* = 0.01, week 2 vs. week 12: *p* = 0.03, week 2 vs. week 14: *p* = 0.01, week 6 vs. week 14: *p* = 0.02; Table 3). We observed a significant increase in frequencies of proliferating Ki67+ CD45RA−CD4 T-cells at the end of treatment (week 0 vs. week 12: *p* = 0.047, Figure 2a). Frequencies of CCR6+ CD45RA−CD4 T-cells, as well as expression of both ectonucleotidases CD39/CD73 by CD4 T-cells, were significantly lower after treatment termination (CCR6: week 1 vs. week14 *p* = 0.02; CD39: week 6 vs. week 14 *p* = 0.04; CD73 week 0 vs. week 14 *p =* 0.02). No significant differences in expression levels of HLA-DR/CD38, CTLA-4, and chemokine receptors CCR4/CXCR3 were observed (Table 3, Figure S1). 

Levels of Th and Treg subsets showed significant alterations after treatment termination. We observed a significant decrease in levels of pro-inflammatory Th17 cells (week 1 vs. week 14: *p* = 0.02) and Th1Th17 cells (week 1 vs. week 14: *p* = 0.04). Frequencies of Th1 showed a significant decrease between weeks 6 and 8 (*p* = 0.04) but with no significant change between the start and end of treatment. Frequencies of Th2 cells decreased significantly at week 6 (week 0 vs. week 6: *p* = 0.04). Lastly, we observed no significant changes in Treg frequency, but their expression levels of CD39+ (week 2 vs. week 12; *p* = 0.008) and CD73+ (week 0 vs. week 14; *p* = 0.008, week 1 vs. week 12, *p* = 0.05) decreased throughout the treatment (Table 3, Figure S2).

#### 3.3.2. Changes in Circulating CD8 T-Cell Subsets 

Significant increases in frequencies of naïve and central memory CD8 T-cells were observed after treatment initiation (naïve: week 0 vs. week 8 *p* = 0.04; central memory: week 1 vs. week 14: *p* = 0.008) with no significant changes in other memory CD8 T-cell subsets over the course of treatment (Table 3). Similar to CD4 T-cells, frequencies of senescent CD8 T-cells showed a significant decrease over the treatment period, reaching statistical significance at week 8 (week 0 vs. week 8: *p* = 0.04). Frequencies of PD-1+CD45RA−CD8 T-cells were also significantly reduced after treatment termination (week 1 vs. week 14: *p* = 0.02). In line with the decrease in CD8 T-cell senescence and exhaustion, we observed an increase in Ki67 expression during the first week of treatment (week 0 vs. week 1: *p* = 0.047). There were no significant changes in frequencies of HLA-DR+CD38+ and CTLA4+CD45RA−CD8 T-cells between the start and the end of treatment (Table 3, Figure S3a,b). 

Within CD45RA−CD8 T-cells, expression levels of CCR6 decreased between weeks 8 and 14 (*p* = 0.02), while levels of CXCR3 increased at week 2 (week 0 vs. week 2: *p* = 0.049) with no overall significant change between the start and the end of treatment. There were no significant alterations in CCR4 over the treatment period. Frequencies of FoxP3+ CD8 T-cells decreased significantly between weeks 2 and 12 (*p* = 0.02). Levels of CD73+ CD8 T-cells increased significantly at week 2 (week 0 vs. week 2: *p* = 0.03). No significant changes in CD39 expression were observed (Table 3, Figure S3c). 

#### 3.3.3. Changes in Monocyte Subsets and Dendritic Cell Frequencies

Levels of classical (CD14++CD16−), intermediate (CD14+CD16+), and non-classical (CD14-CD16++) monocytic subsets remained stable over time (Table 3, Figure S4). Within these subsets, we observed differences in the expression of CD163, CX3CR1, CCR2, and M-DC8 over the treatment duration (Table 3, Figure S5). Expression levels of CD163 in non-classical monocytes showed a significant increase at week 8 (week 0 vs. week 8: *p* = 0.03), followed by a return to baseline after treatment termination (week 6 vs. week 14: *p* = 0.008). Frequencies of CX3CR1+ classical monocytes increased significantly during the first two weeks of treatment (week 0 vs. week 2: *p* = 0.04) but with no overall significant changes before and after treatment course completion. Levels of intermediate and non-classical monocytes expressing CX3CR1 remained stable over time. Expression levels of CCR2 in classical and intermediate monocytes also remained unchanged. In contrast, a significant increase in CCR2+ non-classical monocytes was observed at the end of the treatment period (week 0 vs. week 12: *p* = 0.04, Table 3) with a trend in their fold change increase (Figure 2b). Levels of M-DC8+ cells remained unchanged in non-classical monocytes, whereas in both classical and intermediate monocytes this subpopulation was significantly reduced at the end of treatment (classical: week 1 vs. week 14: *p* = 0.04; intermediate: week 1 vs. week 12: *p* = 0.02, Table 3).

A significant increase in frequencies of myeloid dendritic cells (mDC: CD123−CD11c+), was observed after 12 weeks of oral cannabinoid treatment (week 0 vs. week 12: *p* = 0.04, Table 3) with trend in their fold-change increase (Figure 2c), while no significant changes were observed in levels of plasmacytoid dendritic cells (pDC: CD123+CD11c−) (Table 3, Figure S4). 

### 3.4. Effect of Oral Cannabinoids on Total HIV DNA and Cell-Associated HIV RNA in CD4 T-Cells from Blood and Semen

The impact of oral cannabinoids on HIV reservoir markers was assessed by quantifying total HIV DNA and cell-associated HIV RNA in PBMCs over the treatment period as presented in Table 4. Levels of HIV DNA and cell-associated HIV RNA remained overall stable in circulating CD4 T-cells, although some modest variations were observed between weeks 8 and 12 (Table 4). In only two individuals, HIV DNA was detectable in sperm cells and remained unchanged after study completion. HIV RNA was not detected in the seminal supernatant (not shown). 

## 4. Discussion

To our knowledge, we report for the first time on in vivo anti-inflammatory effects of orally administrated cannabinoid-based treatment in humans in an interventional pilot clinical trial in PWH on ART. Pooled results from eight participants who successfully completed the oral cannabinoid treatment course showed a significant reduction in surrogate markers of gut mucosal damage, systemic inflammation, as well as cellular immune activation, exhaustion, and senescence. These preliminary findings support the use of further evaluation of orally administrated cannabinoid capsules in larger clinical trials as a potential strategy to help alleviate chronic inflammation experienced by PWH despite ART. 

In this pilot clinical trial, our primary objective was the assessment of safety and tolerability, as previously reported [50]. In the current set of analyses, we assessed the effects of oral cannabinoids on the integrity of the gut mucosal barrier by measuring the dynamics of REG-3α and I-FABP over the treatment period. I-FABP is an intracellular protein expressed by enterocytes that is released upon cell death, while REG-3α is an antimicrobial peptide secreted by Paneth cells in the gut lumen that is crucial for the regulation of interplay between the microbiota and the host [65,66]. Notably, our team has previously reported that plasma levels of both markers are known to be elevated in PWH despite ART [7,62]. In the present study, REG-3α plasma levels decreased after 12 weeks of oral cannabinoid treatment. These results are promising since increased gut mucosal permeability is one of the major contributors to chronic inflammation in PWH [5,6]. These findings are also in line with previous observations on the effects of CBD on aspirin-induced gut mucosal permeability where participants treated with CBD and palmitoylethanolamide (an endocannabinoid-like fatty acid) showed significant improvement in gut epithelial integrity [22]. The authors have attributed this effect to the activation of the endocannabinoid system mediated by CBD and the CB1R [22]. Other evidence put forth by in vitro and animal studies also supports the CB1R-mediated reduction in gut epithelial permeability induced by CBD [23,67,68,69,70,71]. It is worth noting that the CB1R signaling cascade is highly complex, where THC acts as a partial CB1R agonist and CBD as a CB1R antagonist [72]. Furthermore, THC and CBD can also bind to other endocannabinoid receptors to exert their anti-inflammatory properties [73]. While we do observe measurable anti-inflammatory effects of oral cannabinoids in our study, the exact mechanism of action remains to be elucidated. 

In our study, plasma levels of LPS and sCD14 showed a transient increase during the first weeks of treatment, followed by a significant reduction later during the treatment course. This observation was unexpected since other groups have reported that CB1R antagonists, such as CBD, reduce plasma LPS levels in diet-induced-obesity models [74]. Cannabidiol-rich cannabis extracts can alter gut microbial composition [75]. Furthermore, diarrhea and abdominal pain are common oral cannabinoid-related adverse effects, indicative of gastrointestinal imbalances that may occur when intake of cannabinoids is first initiated, as it also occurred in our study participants [50]. Furthermore, CBD has been shown to reduce peristalsis via modulation of motor and sensory pathways of the peristaltic reflex [76]. It is plausible that transient gut dysbiosis along with increased retention time of colonic contents may contribute to a temporary increase in LPS absorption that we observe. However, an in-depth analysis of gut microbiome changes over the course of treatment is required to validate this hypothesis. 

Over the treatment course, we also observed a gradual, significant decrease in the sTNFRII inflammatory marker. Interestingly, similar observations on sTNFRII have been reported in PWH after recent cannabis use, although the authors report no change in microbial translocation makers [41]. We also observed a significant reduction in plasma levels of pro-inflammatory cytokines IFN-γ and IL-1β following 12 weeks of treatment which is in line with previous studies that reported a direct inhibitory effect of cannabinoids on pro-inflammatory cytokine production by immune cells in vitro [15] and in animal models [77]. This effect is likely driven by CB2R activation, which is highly expressed in immune cells, including monocytes [16]. 

We further report a significant decrease in frequencies of senescent and PD1+ CD memory T-cells. This was further accompanied by an increase in proliferative memory Ki-67+ CD4 T-cells and lower levels of terminally differentiated CD4 T-cells. Our findings support previous observations made in SIV-infected monkeys, where early THC treatment was associated with decreased levels of PD-1-expressing T-cells [20]. This is of particular importance since T-cell exhaustion and senescence during chronic inflammation, characterized by increased PD1 and CD57 expression, reduced proliferative capacity, and antiviral functions, have been extensively characterized in PWH [5,9,78,79,80]. Tregs are a highly immunosuppressive cell subset that is known to be increased during HIV infection, which has also been linked to disease progression [4,81,82,83,84,85,86]. One way that these cells exert their anti-inflammatory properties is through CD39 and CD73 ectonucleotidases, which convert pro-inflammatory extracellular adenosine triphosphate into immunosuppressive adenosine [87]. Importantly, we previously showed that Tregs-expressing ectonucleotidases are involved in HIV pathogenesis and disease progression, and even early ART initiation could not reverse their increased frequencies and their migration into the gut lymphoid tissues [8,88,89,90]. In our study, we did not observe any significant changes in CD4+ Treg frequencies over the treatment course. However, we do report a significant decrease in CD73+ Tregs, suggesting their decreased immunosuppressive activity. 

Activated monocytes are among the first sources of pro-inflammatory cytokines during HIV infection and are one of the main contributors to chronic inflammatory complications in PWH [6,91]. In our study, we did not observe any changes in intermediate, classical, or non-classical monocytic subsets. Previous studies have reported that cannabis use in HIV-infected individuals is associated with lower levels of inflammatory CD16+ (i.e., intermediate and non-classical) monocytes and lower expression of CD163, indicative of their decreased migration potential toward the brain [42,43]. Notably, those studies looked at cannabis smokers, where THC and CBD were inhaled, whereas in our study these compounds were ingested. It is also known that the bioavailability of inhaled THC and CBD is several fold higher than if they are administered orally [92]. Thus, it is likely that THC/CBD plasma concentrations were insufficient to have a measurable effect on CD16+ monocytes in this clinical trial. However, we did observe a decrease in intermediate M-DC8+ monocytes which are known to be a major source of TNF-α during their response to microbial translocation [91]. Furthermore, oral cannabinoids also significantly increased CCR2-expressing non-classical monocytes, a loss of this subpopulation had been associated with cognitive impairment in PWH not taking ART [93]. The increase in these non-classical monocyte subpopulations during the course of the treatment would be indicative of a potential neuroprotective effect of oral cannabinoids in PWH. Furthermore, we also noticed a significant increase in the frequencies of mDCs during cannabinoid treatment, while the frequency of plasmacytoid dendritic cells remained stable. Although mDCs are well known for promoting the activation and expansion of effector T-cells during inflammatory conditions [94,95], these cells also play an immuno-regulatory role in the maintenance of tissue homeostasis and immune tolerance [94,95]. 

Lastly, given that cannabinoids were reported to be associated with decreased SIV and HIV viral replication [19,48,96,97,98,99], and since an observational study previously reported that cannabis use was associated with intermittent HIV shedding in the semen of men who have sex with men on ART [100], we explored if oral cannabinoids impact HIV reservoir size. While we found no significant changes in HIV DNA/RNA levels neither in blood or semen, larger clinical trials are required to decipher the impact of oral cannabinoids on HIV reservoir dynamics along with the changes in immune cells. 

It is evident that there are several limitations in this pilot clinical trial. First, our initial target sample size included 26 participants randomized in a 1:1 ratio in two arms. Because the production of oral cannabinoid capsules used in this study was permanently discontinued, we were forced to stop the recruitment process prematurely [50]. Furthermore, 2 of the 10 participants were withdrawn from the trial due to adverse events [50]. Thus, unfortunately we lacked the statistical power to compare the different cannabinoid formulations (CBD:THC versus CBD-only), and therefore we had to pool the results of both study arms. However, since each study participant underwent a personalized tolerability titration, and the fact that each study participant was in her/his own control to assess the overtime changes, we believe a nonparametric paired statistical comparison between each two time-points was the only way to analyze our overtime data based on the limited sample size. Potential confounding effects related to the open-label design of this study may also have occurred. Some reports show that an open-label study design has been associated with increased chance of bias and potential overestimation of treatment-related adverse event occurrence rate compared to blinded studies [101,102]. Indeed, since the participants were aware of the formulation they were taking and the possible associated side effects, it might have affected their ability to reach and/or maintain the highest target dose until the end of the treatment period, leading to individual-dependent dosage variations in cannabinoid intake in this study. Moreover, oral cannabinoids have low bioavailability and there is high variability across studies depending on the vehicle of administration [103]. Cannabinoid bioavailability is also heavily impacted by food intake, and consumption of CBD with a high-fat meal can increase its bioavailability [104,105]. Another limitation is the fact that our observations are made on circulating immune cells from peripheral blood, which could underestimate the real effects of cannabinoids on tissue-resident immune cells in the GALT, one of the main sites permanently affected by HIV. Future studies using distal colon pinch biopsies could address the effect of cannabinoids on cytokine and immune cell dynamics in the gastrointestinal tract [106,107]. Finally, we were not able to provide a measure of the replication-competent HIV reservoir. The new intact proviral DNA assay (IPDA) would be a good strategy to measure and discriminate between intact replication-competent, defective, and total HIV genomes. However, we previously showed that total and integrated HIV DNA measures and IPDA measures correlate well with each other [108]. 

## 5. Conclusions

In summary, our findings further support the anti-inflammatory effects of oral cannabinoid capsules in PWH on ART. Oral cannabinoids show promise in improving the gut mucosal barrier, alleviating immune activation, and decreasing T-cell exhaustion and immunosenescence. These findings constitute supplemental evidence of cannabinoids’ therapeutic potential in combination with ART for PWH that could help reduce the rates of non-AIDS-related morbidity and mortality in this population. These findings suggest that larger clinical trials are warranted to further evaluate the role of oral cannabinoids as a strategy to reduce systemic inflammation and gut microbial translocation in PWH on ART. 

## Figures and Tables

**Table 1:** Demographic and biological characteristics of study participants at inclusion.

	Total Population (*n* = 10)
Age (Years), median (±IQR)	57.5 (54.75–61.75)
Sex assigned at birth (*n* (%))	
Male	8 (80%)
Female	2 (20%)
Ethnicity (*n* (%))	
White-North American	6 (60%)
Black-African	1 (10%)
Asian	1 (10%)
Mixed ethnicity	2 (20%)
Antiretroviral regimens (*n* (%))	
Biktarvy^®^ (Bictegravir/Tenofovir alafenamide/Emtricitabine)	5 (50%)
Triumeq^®^ (Dolutegravir/Abacavir/Lamivudine)	1 (10%)
Truvada^®^/Viramure^®^ (Tenofovir/Emtricitabine/Nevirapine)	1 (10%)
Raltegravir/Kivexa^®^/Biktarvy^®^ (Raltegravir/Abacavir/Lamivudine/Bictegravir/Tenofovir alafenamide/Emtricitabine)	1 (10%)
Genvoya^®^ (Elvitegravir/Cobicistat/Emtricitabine/Tenofovir alafenamide)	1 (10%)
Delstrigo^®^ (Doravirine/Lamivudine/Tenofovir disoproxil fumarate)	1 (10%)
Oral cannabinoids regimens (*n* (%))	
TN-TC11M2 formulation (CBD: 2.5/THC: 2.5 mg)	5 (50%)
TN-C200M2 formulation (CBD: 200 mg)	5 (50%)
Cannabis use in the past 6 months before study initiation (*n* (%))	
No	3 (30%)
Yes	7 (70%)
Monthly	5 (72.43%)
Weekly	2 (28.57%)
Daily	0 (0%)
Alcohol use in the past 6 months (*n* (%))	
No	5 (50%)
Yes	5 (50%)
Drug use in the past 6 months (*n* (%))	
No	3 (30%)
Yes	7 (70%)
History of infectious diseases (*n* (%))	
Syphilis (treated)	2 (20%)
Hepatitis B (Anti-HBc Antibodies)	4 (40%)
Hepatitis C (Anti-HCV Antibodies)	0 (0%)

**Table 2:** Dynamic of plasma soluble markers of gut mucosal damages (REG-3α, I-FABP), microbial translocation and immune activation (LPS, sCD14, and sCD27, sTNFRII), and pro-inflammatory (TNF-α, IFN-γ, IL-1β, IL-6, IL-8, IP-10) and anti-inflammatory (IL-10) cytokines during cannabinoids treatment, from treatment initiation to study termination.

Plasma Markers	Study Time-Line						
	Week 0 Treatment Initiation *n* = 10	Week 1 *n* = 10	Week 2 *n* = 10	^$^ Week 6 *n* = 9	^$^ Week 8 *n* = 8	^$^ Week 12 End of Treatment *n* = 8	^$^ Week 14 Study Termination *n* = 8
Gut mucosal damage							
REG-3α (pg/mL) [Mean (SD)]	**5621 (3299) ^b,e^**	**5390 (3294) ^g^**	**4663 (2970) ^b,g,l,m^**	**5610 (3403) ^l,q,r^**	**5315 (3140) ^m,s,t^**	**4950 (3018) ^e,q,s^**	**4757 (3366) ^r,t^**
[Median (IQR)]	**5608 (2617–7039) ^b,e^**	**5157 (2533–6751) ^g^**	**4893 (2220–5740) ^b,g,l,m^**	**5620 (2582- 6762) ^l,q,r^**	**5784 (2451–6430) ^m,s,t^**	**5612 (2074–6013) ^e,q,s^**	**4834 (1705–6048) ^r,t^**
I-FABP (pg/mL) [Mean (SD)]	895.6 (774.2)	1129 (1026)	1117 (1160)	794.5 (455.6)	887.4 (768.6)	802.3 (581.7)	709.3 (526.4)
[Median (IQR)]	660.6 (436.6–1275)	731.1 (422.2–2001)	717.8 (549.3–1174)	557.5 (354.6–1202)	665.8 (316.6–1368)	658.0 (288.6–1338)	517.6 (388.1–916.1)
Microbial translocation and immune activation							
LPS (pg/mL) [Mean (SD)]	**98.7 (47.76) ^d^**	**99.75 (54.67) ^i^**	**89.3 (45.11) ^m^**	**104.7 (43.97) ^p^**	**138.3 (63.09) ^d,i,m,p,s,t^**	**112.2 (45.7) ^s^**	**94.73 (37.82) ^t^**
[Median (IQR)]	**88.29 (60.6–144.0) ^d^**	**93.18 (55.7–139.7) ^i^**	**76.56 (56.2–142.3) ^m^**	**102.8 (64.8–146.4) ^p^**	**131.9 (98.8–197.7) ^d,i,m,p,s,t^**	**115.8 (80.7–136.2) ^s^**	**98.78 (72.6–125.4) ^t^**
sCD14 (ng/mL) [Mean (SD)]	2583 (536.9)	**2446 (447.3) ^h^**	2605 (472.9)	**2855 (676.3) ^h,p,q^**	**2407 (399.2) ^p^**	**2491 (449.7) ^q^**	2650 (602.7)
[Median (IQR)]	2472 (2166–2825)	**2344 (2110–2877) ^h^**	2493 (2248–2995)	**2766 (2371–3257) ^h,p,q^**	**2327 (2034–2797) ^p^**	**2371 (2181–2800) ^q^**	2523 (2144–2945)
sCD27 (U/mL) [Mean (SD)]	110.9 (22.73)	113.3 (31.55)	111.4 (26.81)	113.3 (28.88)	112.8 (35.98)	109.3 (35.76)	108.6 (28.85)
[Median (IQR)]	114.6 (87.3–127.2)	110.5 (93.4–122.3)	105.7 (88.2–135.2)	103.4 (88.8–137.8)	108.6 (79.5–136.2)	91.20 (83.5–141.9)	106.3 (80.6–132.8)
sTNFRII (pg/mL) [Mean (SD)]	**2297 (665.4) ^d,e^**	**2279 (713.7) ^i,j^**	**2329 (800.1) ^n^**	2631 (1076)	**2096 (481.1) ^d,i^**	**2027 (410.0) ^e,j,n^**	2215 (740.8)
[Median (IQR)]	**2202 (1708–2823) ^d,e^**	**2037 (1680–3061) ^i,j^**	**2115 (1639–3158) ^n^**	2608 (1752–3321)	**1971 (1727–2626) ^d,i^**	**1938 (1674–2353) ^e,j,n^**	1957 (1719–3002)
Pro-inflammatory cytokines							
TNF-α (pg/mL) [Mean (SD)]	**2.61 (1.64) ^f^**	2.18 (1.19)	**2.57 (2.17) ^o^**	2.06 (1.10)	2.27 (0.77)	2.03(0.71)	**2.13 (1.48) ^f,o^**
[Median (IQR)]	**2.38 (1.38–3.63) ^f^**	2.16 (0.87–3.21)	**1.82 (0.89–3.58) ^o^**	1.93 (1.27–2.69)	2.09 (1.54–2.91)	1.94 (1.39–2.67)	**1.56 (0.95–3.78) ^f,o^**
INF-γ (pg/mL) [Mean (SD)]	**10.98 (18.11) ^e^**	8.24 (11.43)	14.31 (24.62)	**9.9 (13.52) ^q^**	9.26 (11.73)	**8.55 (11.63) ^e,q^**	9.93 (15.35)
[Median (IQR)]	**3.38 (0.53–13.63) ^e^**	3.2 (0.53–13.28)	2.28 (0.48–21.01)	**1.63 (0.52–21.62) ^q^**	2.79 (0.54–18.82)	**3.32 (0.54–20.81) ^e,q^**	2.66 (0.67–21.74)
IL-1β (pg/mL) [Mean (SD)]	0.52 (0.45)	**0.62 (0.64) ^h,j^**	0.58 (0.58)	**0.43 (0.34) ^h^**	0.40 (0.20)	**0.40 (0.24) ^j^**	0.62 (0.72)
[Median (IQR)]	0.44 (0.24–0.59)	**0.47 (0.18–0.68) ^h,j^**	0.46 (0.26–0.59)	**0.38 (0.14–0.65) ^h^**	0.46 (0.17–0.60)	**0.44 (0.15–0.52) ^j^**	0.42 (0.19–0.66)
IL-6 (pg/mL) [Mean (SD)]	1.44 (1.00)	1.11 (0.75)	1.36 (0.97)	1.36 (0.91)	1.40 (1.15)	1.22 (0.97)	1.29 (1.13)
[Median (IQR)]	1.14 (0.51–2.30)	0.91 (0.41–1.91)	1.30 (0.55–1.86)	0.92 (0.63–2.30)	0.87(0.63–1.92)	0.88 (0.47–1.69)	1.07 (0.38–1.72)
IL-8 (pg/mL) [Mean (SD)]	5.50 (3.5)	5.79 (5.6)	5.12 (3.18)	5.53 (2.88)	**6.20 (2.41) ^t^**	5.61 (2.99)	**4.21 (1.42) ^t^**
[Median (IQR)]	4.97 (3.05–7.38)	3.88 (2.08–8.10)	4.89 (2.80–7.67)	5.94 (2.74–7.92)	**5.84 (4.00–8.37) ^t^**	5.76 (2.66–7.59)	**4.68 (2.86–5.32) ^t^**
IP-10 (pg/mL) [Mean (SD)]	55.99 (36.72)	**55.5 (33.47) ^h^**	51.26 (24.24)	**50.55 (26.04) ^h,p^**	**61.80 (35.97) ^p^**	56.68 (25.52)	52.59 (28.58)
[Median (IQR)]	43.74 (30.11–77.91)	**43.18 (31.62–76.80) ^h^**	42.97 (32.30–74.06)	**41.51 (35.77–69.95) ^h,p^**	**50.05 (36.14–95.64) ^p^**	48.65 (42.49–81.62)	41.80 (33.77–75.17)
Anti-inflammatory cytokine							
IL-10 (pg/mL) [Mean (SD)]	1.26 (1.37)	0.92 (0.54)	1.0 (0.68)	0.93 (0.44)	1.1 (0.85)	1.1 (0.74)	0.93 (0.49)
[Median (IQR)]	0.77 (0.49–1.55)	0.79 (0.51–1.28)	0.85 (0.47–1.26)	0.86 (0.53–1.29)	0.82 (0.50–1.36)	0.87 (0.60–1.52)	0.90 (0.52–1.18)

Results are shown as the mean and standard deviation (SD) and as the median and interquartile range (IQR). Significant differences (*p* < 0.05) following the Wilcoxon matched-pairs signed-rank test are mentioned as follows: ^a^: week 0 vs. week 1; ^b^: week 0 vs. week 2; ^c^: week 0 vs. week 6; ^d^: week 0 vs. week 8; ^e^: week 0 vs. week 12; ^f^: week 0 vs. week 14; ^g^: week 1 vs. week 2; ^h^: week 1 vs. week 6; ^i^: week 1 vs. week 8; ^j^: week 1 vs. week 12; ^k^: week 1 vs. week 14; ^l^: week 2 vs. week 6; ^m^: week 2 vs. week 8; ^n^: week 2 vs. week 12; ^o^: week 2 vs. week 14; ^p^: week 6 vs. week 8; ^q^: week 6 vs. week 12; ^r^: week 6 vs. week 14; ^s^: week 8 vs. week 12; ^t^: week 8 vs. week 14; ^u^: week 12 vs. week 14. Significant values are presented in bold. ^$^: Two participants from CBD-only were excluded from analyses at week 6 (one participant) and weeks 8, 12, and 14 (two participants) because they were withdrawn for safety concerns.

**Table 3:** Dynamic of CD4 and CD8 T-cells, monocytes and dendritic cells populations during cannabinoids treatment.

Cellular Immune Markers	Study Time-Line						
	Week 0 Treatment Initiation *n* = 10	Week 1 *n* = 10	Week 2 *n* = 10	^$^ Week 6 *n* = 9	^$^ Week 8 *n* = 8	^$^ Week 12 End of Treatment *n* = 8	^$^ Week 14 Study Termination *n* = 8
CD4 T-Cells							
Memory T-Cell Subsets							
Naïve (CD45RA+CD28+CCR7+) % [Mean (SD)]	78.2 (13.1)	79.6 (11.0)	79.4 (12.5)	78.0 (14.3)	76.7 (13.5)	76.2 (13.6)	73.9 (15.6)
[Median (IQR)]	83.4 (66.3–87.4)	83.8 (74.2–86.6)	82.0 (74.4–87.4)	86.2 (65.4–87.7)	81.9 (63.1–85)	80.7 (63.9–84.8)	78.7 (59.1–85.4)
Central Memory (CD45RA−CD28+CCR7+) % [Mean (SD)]	32.9 (11.7)	33.6 (10.6)	35.3 (12.7)	34.4 (12.7)	32.5 (11.5)	32.7 (12.8)	32.7 (12.4)
[Median (IQR)]	36.6 (22.3–39.6)	33.3 (28.3–40.1)	34.5 (29.3–42.2)	34.4 (25.8–44.1)	34.7 (24.8–38.6)	34.5 (21.1–40.9)	33.8 (22.8–39.7)
Transitional Memory (CD45RA−CD28+CCR7−) % [Mean (SD)]	64.1 (11.1)	63.8 (10.6)	62.4 (12.5)	63.0 (12.6)	64.9 (11.4)	64.4 (12.7)	63.6 (13.3)
[Median (IQR)]	62 (57.2–71.7)	63.4 (57.6–69.6)	62.6 (52.2–68.3)	60.9 (52.2–73.2)	63.6 (56.6–73.0)	61.5 (55.3–77.4)	62.3 (50.7–75.7)
Effector Memory (CD45RA−CD28−CCR7−) % [Mean (SD)]	**2.6 (3.2) ^c^**	**2.3 (2.8) ^i^**	2.1 (2.5)	**2.3 (2.7) ^c^**	**2.4 (2.7) ^i^**	2.6 (2.3)	3.2 (3.9)
[Median (IQR)]	**1.0 (0.3–5.9) ^c^**	**1.0 (0.4–4.6) ^i^**	1.1 (0.3–3.7)	**0.8 (0.3–4.5) ^c^**	**1.2 (0.4–5.1) ^i^**	1.4 (0.8–5.3)	1.6 (0.5–5.6)
Terminally Differentiated (CD45RA+CD28−CCR7−) % [Mean (SD)]	**2.1 (4.4) ^b^**	**2.1 (4.6) ^g,i^**	**1.6 (3.9) ^b,g^**	2.1 (4.4)	**1.8 (4.2) ^i^**	1.8 (3.6)	2.4 (5.6)
[Median (IQR)]	**0.2 (0.04–1.9) ^b^**	**0.2 (0.04–1.6) ^g,i^**	**0.1 (0.03–1.1) ^b,g^**	0.2 (0.05–2.2)	**0.1 (0.09–1.3) ^i^**	0.2 (0.07–1.8)	0.1 (0.05–1.8)
T-cell functions							
HLADR+CD38+ (%) [Mean (SD)]	3.1 (1.6)	4.0 (3.1)	3.8 (4.1)	2.8 (1.7)	2.7 (1.7)	3.6 (2.7)	3.2 (3.6)
[Median (IQR)]	2.9 (1.6–4.8)	3.8 (1.6–4.9)	2.3 (1.6–4.6)	1.7 (1.3–4.4)	2.2 (1.5–4.6)	2.5 (1.3–6.5)	1.8 (1.2–4.2)
CD45RA−Ki-67+ (%) [Mean (SD)]	**0.8 (0.3) ^e^**	0.9 (0.4)	0.9 (0.4)	1.0 (0.5)	1.0 (0.6)	**1.0 (0.4) ^e^**	0.8 (0.3)
[Median (IQR)]	**0.7 (0.5–1.0) ^e^**	0.8 (0.5–1.3)	0.8 (0.5–1.2)	1.0 (0.6–1.2)	0.8 (0.6–1.2)	**0.9 (0.7–1.2) ^e^**	0.7 (0.7–0.9)
CD45RA−PD-1+ (%) [Mean (SD)]	**32.3 (9.5) ^f^**	**32.4 (9.5) ^j,k^**	**31.4 (9.5) ^n,o^**	**32.2 (10.1) ^r^**	32.6 (10.3)	**30.6 (11.0) ^j,n^**	**30.8 (9.2) ^f,k,o,r^**
[Median (IQR)]	**30.5 (26.3–39.0) ^f^**	**30.7 (24.9–38.3) ^j,k^**	**30.6 (23.8–37.5) ^n,o^**	**32.2 (23.0–37.8) ^r^**	31.2 (24.5–41.7)	**31.3 (22.6–39.2) ^j,n^**	**30.5 (22.6–37.8) ^f,k,o,r^**
CD45RA−CTLA-4+ (%) [Mean (SD)]	3.0 (1.4)	3.2 (1.5)	3.3 (1.7)	3.6 (2.4)	3.9 (2.2)	3.6 (1.4)	3.8 (1.6)
[Median (IQR)]	3.1 (1.8–4.2)	2.5 (2.0–4.9)	2.5 (2.0–4.9)	3.2 (1.7–5.0)	3.2 (2.4–5.4)	3.8 (2.3–5.1)	3.9 (2.2–4.7)
Senescent (CD28−CD57+) (%) [Mean (SD)]	1.8 (2.5)	**1.5 (2.1) ^i^**	1.3 (1.7)	1.5 (2.0)	**1.4 (1.6) ^i^**	1.3 (1.2)	2.1 (3.2)
[Median (IQR)]	0.5 (0.07–4.0)	**0.7 (0.05–3.0) ^i^**	0.7 (0.04–2.3)	0.7 (0.1–2.6)	**0.7 (0.2–2.2) ^i^**	0.8 (0.5–2.0)	0.9 (0.3–2.2)
CD4+CD39+ (%) [Mean (SD)]	6.1 (4.7)	**6.3 (5.1) ^g^**	**5.9 (4.8) ^g^**	**6.4 (5.1) ^r^**	**6.4 (5.3) ^t^**	5.9 (5.7)	**5.5 (4.8) ^r,t^**
[Median (IQR)]	5.1 (2.1–9.0)	**5.0 (1.9–9.0) ^g^**	**4.9 (1.8–8.1) ^g^**	**5.8 (1.5–10.3) ^r^**	**5.0 (1.8–11.4) ^t^**	5.1 (1.3–7.9)	**4.8 (1.3–7.8) ^r,t^**
CD4+CD73+ (%) [Mean (SD)]	**6.9 (3.1) ^d,f^**	6.4 (2.6)	**6.6 (2.8) ^o^**	6.2 (3.0)	**6.1 (2.8) ^d^**	6.3 (3.7)	**5.8 (2.9) ^f,o^**
[Median (IQR)]	**7.1 (4.6–9.2) ^d,f^**	6.8 (4.5–7.9)	**6.5 (4.9–7.9) ^o^**	6.2 (3.9–7.3)	**6.6 (3.6–7.0) ^d^**	6.1 (3.4–6.8)	**5.6 (3.3–6.9) ^f,o^**
Chemokine receptors expression							
CD45RA−CCR4+ (%)[Mean (SD)]	29.7 (11.4)	29.2 (10.2)	30.1 (10.8)	31.4 (9.5)	30.5 (9.8)	29.4 (10.1)	29.1 (10.4)
[Median (IQR)]	31 (20.5–36.6)	31.6 (21.1–36.0)	31.7 (20.8–38.0)	34.4 (23.2–36.1)	31.8 (20.3–36.6)	30.1 (18.6–38.4)	30.6 (18.3–38.1)
CD45RA−CCR6+ (%) [Mean (SD)]	9.8 (6.1)	**12.6 (7.1) ^k^**	11.7 (5.0)	15.0 (10.8)	12.0 (7.2)	10.9 (4.3)	**9.7 (6.4) ^k^**
[Median (IQR)]	8.8 (5.7–12.4)	**11.8 (7.1–19.0) ^k^**	11.2 (8.3–16.2)	10.6 (7.4–20.8)	11.4 (8.3–13.3)	10.3 (8.5–13.2)	**6.7 (5.7–15.5) ^k^**
CD45RA−CXCR3+ (%) [Mean (SD)]	2.6 (2.9)	3.8 (5.2)	4.1 (3.8)	5.7 (3.6)	2.4 (3.1)	3.5 (3.2)	3.1 (4.7)
[Median (IQR)]	1.7 (0.3–4.1)	2.4 (0.4–4.1)	3.0 (1.8–6.1)	4.9 (3.0–9.1)	0.8 (0.5–5.2)	2.3 (1.7–5.3)	1.2 (0.9–3.5)
Th subsets							
Th17 (CD45RA−CCR4+CCR6+ CXCR3−) (%) [Mean (SD)]	5.2 (3.0)	**6.8 (4.0) ^k^**	6.4 (2.8)	8.0 (5.9)	6.3 (3.6)	5.8 (2.5)	**5.2 (3.5) ^k^**
[Median (IQR)]	5.2 (2.8–7.2)	**5.5 (4.4–10.3) ^k^**	6.0 (4.8–9.2)	6.9 (3.4–10.7)	5.7 (4.8–7.1)	5.4 (5.0–6.7)	**3.8 (2.4–8.2) ^k^**
Th1-Th17 (CD45RA−CCR4−CCR6+CXCR3+) (%) [Mean (SD)]	**0.36 (0.6) ^c^**	**0.62 (0.9) ^k^**	0.6 (0.9)	**1.0 (1.1) ^c^**	0.5 (0.9)	0.3 (0.3)	**0.2 (0.3) ^k^**
[Median (IQR)]	**0.2 (0.02–0.4) ^c^**	**0.3 (0.05–0.8) ^k^**	0.3 (0.1–0.7)	**0.4 (0.3–1.7) ^c^**	0.06 (0.03–0.9)	0.2 (0.1–0.4)	**0.1 (0.04–0.2) ^k^**
Th2 (CD45RA−CCR4+CCR6−CXCR3−) (%) [Mean (SD)]	**23.0 (10.5) ^c^**	20.7 (7.6)	21.9 (8.9)	**20.9 (6.5) ^c^**	22.9 (10.4)	22.1 (8.9)	22.6 (8.5)
[Median (IQR)]	**21.9 (13.7–32.6) ^c^**	20.0 (14.6–29.1)	20.7 (14.7–30.7)	**20.9 (14.7–26.9) ^c^**	21.7 (13.4–31.9)	20.9 (14.0–30.0)	23.7 (13.6–29.6)
Th1 (CD45RA−CCR4−CCR6−CXCR3+) (%) [Mean (SD)]	1.7 (1.7)	2.4 (3.6)	2.7 (2.5)	3.2 (1.8) **^p^**	1.4 (1.6) **^p^**	2.5 (2.3)	2.4 (3.8)
[Median (IQR)]	1.3 (0.3–3.0)	1.3 (0.3–2.5)	2.0 (1.0–4.5)	3.6 (1.9–4.7) **^p^**	0.6 (0.4–2.9) **^p^**	1.8 (1.0–4.4)	0.9 (0.7–2.5)
Regulatory T-cells							
Treg (CD25hi CD127lo FoxP3+) (%) [Mean (SD)]	2.9 (1.6)	2.7 (1.4)	2.9 (1.2)	3.2 (1.6)	3.4 (1.7)	2.6 (1.1)	2.9 (1.1)
[Median (IQR)]	2.0 (1.5–4.5)	2.6 (1.5–3.6)	2.5 (1.9–3.9)	2.7 (1.8–4.4)	3.7 (1.6–4.8)	2.0 (1.8–3.8)	2.6 (2.2–4.1)
CD73+ Treg (%) [Mean (SD)]	**5.0 (2.4) ^f^**	**4.9 (2.6) ^j,k^**	4.8 (2.5)	4.2 (2.2)	4.5 (2.1)	**4.2 (1.7) ^j^**	**4.2 (1.7) ^f,k^**
[Median (IQR)]	**4.9 (3.0–7.4) ^f^**	**4.1 (3.3–7.3) ^j,k^**	4.4 (2.9–7.2)	3.5 (3.0–5.8)	4.3 (3.0–6.6)	**3.9 (3.5–5.7) ^j^**	**3.9 (3.4–5.9) ^f,k^**
CD39+ Treg (%) [Mean (SD)]	45.1 (21.1)	45.4 (23.0)	**44.0 (22.2) ^n^**	44.1 (24.5)	41.3 (23.3)	**41.3 (21.7) ^n^**	41.0 (20.4)
[Median (IQR)]	41.5 (28.5–62.5)	46.3 (23.4–65.6)	**43.4 (25.2–65.4) ^n^**	43.2 (24.1–69.2)	36.5 (21.5–66.6)	**37.4 (23.3–59.8) ^n^**	38.1 (25.1–63.3)
CD8 T-cells							
Memory T-cell subsets							
Naïve (CD45RA+CD28+CCR7+) % [Mean (SD)]	**50.4 (25.3) ^b,d^**	**52.3 (24.7) ^i^**	**54.6 (25.3) ^b^**	51.8 (24.7)	**52.8 (26.8) ^d,i^**	48.2 (25.9)	52.8 (25.6)
[Median (IQR)]	**48.7 (24.4–77.7) ^b,d^**	**53.4 (26.5–77.4) ^i^**	**55.3 (29.8–80.1) ^b^**	51.2 (27.4–74.2)	**57.5 (25.9–77.7) ^d,i^**	47.0 (24.0–67.5)	56.2 (28.9–77.8)
Central Memory (CD45RA−CD28+CCR7+) % [Mean (SD)]	9.7 (6.1)	**10.0 (4.7) ^k^**	10.8 (5.3)	12.1 (6.3)	11.8 (4.5)	11.5 (4.5)	**12.1 (5.3) ^k^**
[Median (IQR)]	7.6 (4.9–15.9)	**8.6 (5.7–14.6) ^k^**	8.2 (6.5–17.4)	9.5 (5.8–18.4)	12.9 (6.8–16.0)	10.8 (7.3–15.1)	**12.6 (6.4–17.6) ^k^**
Transitional Memory (CD45RA−CD28+CCR7−) % [Mean (SD)]	66.7 (12.0)	67.3 (11.1)	67.5 (10.7)	65.2 (9.7)	67.4 (9.5)	66.3 (8.1)	64.7 (13.1)
[Median (IQR)]	70.5 (58.7–75.2)	72.0 (58.5–74.7)	69.4 (58.0–74.7)	69.2 (59.0–72.8)	72.1 (61.3–73.6)	70.7 (59.2–72.0)	70.6 (57.0–72.5)
Effector Memory (CD45RA−CD28−CCR7−)% [Mean (SD)]	22.8 (15.6)	21.9 (13.9)	21.0 (13.6)	21.7 (14.2)	19.8 (12.8)	21.2 (10.6)	21.8 (15.8)
[Median (IQR)]	17.1 (9.8–35.7)	15.6 (9.8–33.8)	14.8 (9.3–34.5)	19.2 (7.9–32.2)	14.1 (9.9–30.9)	16.9 (13.3–33.4)	14.1 (10.7–35.3)
Terminally Differentiated (CD45RA+CD28−CCR7−) % [Mean (SD)]	31.2 (22.2)	30.0 (22.7)	28.5 (22.0)	31.4 (21.6)	29.8 (23.4)	33.0 (23.9)	29.6 (22.5)
[Median (IQR)]	35.5 (5.7–49.3)	32.1 (4.4–54.1)	29.5 (4.2–51.6)	30.6 (9.7–53.1)	26.5 (6.4–55.4)	38.4 (6.5–55.4)	28.6 (6.9–48.2)
T-cell functions							
HLA-DR+CD38+ (%) [Mean (SD)]	4.9 (4.1)	5.3 (4.3)	5.2 (5.0)	5.1 (3.8)	6.1 (6.7)	6.1 (5.7)	5.4 (5.0)
[Median (IQR)]	3.6 (2.0–7.4)	4.3 (2.6–6.9)	3.5 (1.6–8.2)	4.4 (2.2–7.8)	3.5 (1.8–9.0)	3.4 (2.7–11.1)	3.8 (2.1–8.5)
CD45RA−Ki-67+ (%) [Mean (SD)]	**0.39 (0.19) ^a^**	**0.51 (0.25) ^a^**	0.49 (0.27)	0.55 (0.40)	0.58 (0.63)	0.52 (0.18)	0.58 (0.46)
[Median (IQR)]	**0.41 (0.25–0.56) ^a^**	**0.47 (0.33–0.73) ^a^**	0.47 (0.32–0.74)	0.42 (0.35–0.54)	0.36 (0.28–0.49)	0.53 (0.34–0.68)	0.36 (0.30–0.96)
CD45RA−PD-1+ (%) [Mean (SD)]	37.9 (13.1)	**37.9 (11.9) ^k^**	37.7 (14.9)	35.2 (10.8)	**37.1 (11.6) ^t^**	34.9 (12.8)	**34.7 (10.2) ^k,t^**
[Median (IQR)]	37.0 (31.0–45.3)	**38.1 (29.5–46.5) ^k^**	35.3 (27.5–47.8)	35.9 (27.1–45.7)	**36.4 (29.4–49.2) ^t^**	37.2 (25.1–47.1)	**36.7 (27.2–42.9) ^k,t^**
CD45RA−CTLA-4+ (%) [Mean (SD)]	1.0 (0.7)	1.3 (1.0)	1.4 (0.9)	2.1 (3.1)	1.1 (1.0)	1.0 (0.6)	1.3 (0.9)
[Median (IQR)]	0.9 (0.5–1.2)	1.0 (0.6–1.7)	1.2 (0.6–2.2)	0.8 (0.5–2.7)	0.9 (0.4–1.4)	1.0 (0.6–1.1)	1.0 (0.7–2.0)
Senescent (CD28−CD57+) (%) [Mean (SD)]	**18.5 (14.5) ^c,d^**	17.0 (13.5)	**16.4 (12.6) ^m^**	**17.9 (13.5) ^c^**	**16.2 (12.5) ^d,m^**	17.0 (11.1)	18.1 (15.4)
[Median (IQR)]	**16.1 (4.9–31.8) ^c,d^**	13.6 (4.5–31.4)	**12.5 (4.6–29.6) ^m^**	**16.8 (5.7–31.6) ^c^**	**10.6 (6.4–27.7) ^d,m^**	14.9 (5.8–28.2)	11.4 (7.2–29.9)
CD8+CD39+ (%) [Mean (SD)]	2.3 (1.4)	2.6 (1.9)	2.5 (1.9)	**2.6 (2.2) ^r^**	2.3 (1.9)	2.2 (1.6)	**2.0 (1.6) ^r^**
[Median (IQR)]	2.8 (0.5–3.4)	2.6 (0.6–3.9)	2.7 (0.4–3.8)	**2.8 (0.3–4.5) ^r^**	2.1 (0.4–3.8)	2.1 (0.6–3.4)	**2.2 (0.3–3.3) ^r^**
CD8+CD73+ (%) [Mean (SD)]	**36.6 (24.4) ^b^**	36.9 (24.6)	**38.1 (24.7) ^b^**	32.9 (19.8)	32.9 (20.7)	30.9 (21.4)	32.1 (21.3)
[Median (IQR)]	**35.3 (10.4–54.4) ^b^**	35.8 (10.5–54.3)	**39.5 (11.7–55.6) ^b^**	36.6 (10.8–50.2)	36.6 (10.8–51.8)	30.6 (9.2–52.9)	34.8 (9.3–50.1)
CD8+FoxP3+ (%) [Mean (SD)]	0.29 (0.23)	0.28 (0.19)	**0.40 (0.29) ^n^**	**0.39 (0.35) ^q^**	0.42 (0.51)	**0.20 (0.19) ^n,q^**	0.28 (0.16)
[Median (IQR)]	0.19 (0.13–0.56)	0.21 (0.17–0.35)	**0.37 (0.12–0.60) ^n^**	**0.29 (0.11–0.67) ^q^**	0.14 (0.08–0.80)	**0.12 (0.09–0.29) ^n,q^**	0.25 (0.13–0.44)
Chemokine receptors expression							
CD45RA−CCR4+ (%) [Mean (SD)]	15.8 (10.6)	15.2 (7.5)	15.6 (8.4)	18.2 (10.3)	17.6 (9.5)	17.7 (11.4)	18.2 (9.3)
[Median (IQR)]	13.7 (8.1–21.9)	13.2 (8.6–21.6)	13.9 (9.0–20.9)	15.5 (8.1–28.8)	17.2 (10.2–26.8)	14.1 (9.4–31.0)	17.0 (9.6–26.0)
CD45RA−CCR6+ (%) [Mean (SD)]	4.4 (4.2)	4.9 (3.8)	4.5 (3.0)	5.3 (4.2)	**5.1 (4.4) ^t^**	4.0 (2.4)	**4.0 (3.8) ^t^**
[Median (IQR)]	3.8 (1.4–5.5)	3.5 (1.6–6.7)	3.0 (2.1–7.5)	4.5 (2.4–6.8)	**3.9 (2.1–6.2) ^t^**	3.2 (2.5–5.7)	**3.1 (1.9–3.8) ^t^**
CD45RA−CXCR3+ (%) [Mean (SD)]	**4.1 (3.1) ^b^**	6.0 (4.7)	**7.1 (5.5) ^b^**	9.3 (6.4)	4.7 (4.1)	6.4 (5.2)	5.9 (4.2)
[Median (IQR)]	**4.0 (1.5–6.3) ^b^**	5.1 (1.8–9.2)	**5.6 (3.2–11.8) ^b^**	8.7 (3.7–14.7)	2.7 (2.1–8.6)	4.6 (2.8–10.3)	3.9 (2.7–10.0)
Monocytes							
Classical monocytes							
Classical (CD14++CD16−) (%) [Mean (SD)]	78.0 (13.0)	78.2 (11.8)	77.5 (10.8)	75.1 (16.7)	74.9 (14.5)	74.7 (14.6)	73.0 (16.0)
[Median (IQR)]	78.6 (69.7–90.9)	80.3 (65.4–86.2)	80.4 (65.5–87.1)	83.5 (57.6–89.2)	77.1 (58.4–89.1)	69.2 (62.6–91.8)	80.4 (59.9–86.1)
Classical CD163+ (%) [Mean (SD)]	46.4 (19.2)	44.7 (23.3)	54.1 (26.7)	50.8 (19.3)	**56.0 (20.4) ^t^**	43.5 (19.0)	**46.1 (21.0) ^t^**
[Median (IQR)]	40.0 (32.0–62.2)	39.3 (31.6–64.8)	56.5 (30.3–79.1)	48.2 (33.8–62.1)	**57.2 (41.0–73.5) ^t^**	49.2 (24.7–53.3)	**41.1 (32.0–64.0) ^t^**
Classical CX3CR1+ (%) [Mean (SD)]	**51.2 (14.3) ^b^**	52.7 (14.4)	**60.3 (15.8) ^b^**	51.5 (13.4)	52.3 (11.9)	52.1 (11.9)	55.7 (12.9)
[Median (IQR)]	**53.8 (38.3–61.3) ^b^**	56.6 (40.8–62.3)	**59.7 (54.3–69.0) ^b^**	56.5 (38.1–63.1)	51.9 (44.8–64.4)	56.4 (41.6–61.8)	57.4 (47.1–66.7)
Classical M-DC8+ (%) [Mean (SD)]	2.56 (3.72)	**3.24 (6.73) ^k^**	**3.53 (6.94) ^o^**	1.61 (2.00)	**3.18 (6.28) ^t^**	2.11 (2.93)	**2.64 (5.78) ^k,o,t^**
[Median (IQR)]	0.61 (0.42–4.26)	**0.75 (0.48–2.62) ^k^**	**0.64 (0.56–3.90) ^o^**	0.72 (0.41–2.31)	**0.74 (0.46–2.42) ^t^**	0.61 (0.38–4.86)	**0.49 (0.33–1.43) ^k,o,t^**
Classical CCR2+ (%) [Mean (SD)]	91.6 (3.1)	92.6 (2.6)	92.9 (3.2)	92.1 (3.6)	93.5 (2.9)	92.5 (4.6)	92.2 (5.7)
[Median (IQR)]	92.3 (89.9–93.3)	92.3 (90.8–94.8)	93.4 (91.1–95.7)	91.1 (89.7–95.0)	94.0 (91.6–95.1)	93.4 (91.0–95.2)	94.4 (89.0–95.3)
Intermediate monocytes							
Intermediate (CD14+CD16+) (%) [Mean (SD)]	11.0 (4.9)	10.4 (4.6)	10.5 (4.4)	12.0 (7.2)	12.0 (5.0)	12.9 (6.7)	12.7 (5.7)
[Median (IQR)]	11.0 (6.0–15.3)	10.5 (6.4–14.9)	12.0 (6.2–13.7)	9.1 (6.5–21.0)	14.3 (6.4–15.2)	14.4 (5.5–18.9)	11.5 (8.1–18.0)
Intermediate CD163+ (%) [Mean (SD)]	66.2 (11.7)	66.7 (15.5)	74.0 (17.6)	70.4 (12.5)	**73.5 (12.4) ^t^**	64.5 (14.2)	**65.9 (15.0) ^t^**
[Median (IQR)]	65.2 (59.4–72.2)	69.7 (58.1–76.2)	74.0 (60.4–88.1)	69.4 (60.3–77.5)	**71.4 (64.9–84.2) ^t^**	59.6 (54.2–78.9)	**64.7 (54.6–78.6) ^t^**
Intermediate CX3CR1+ (%) [Mean (SD)]	86.1 (8.2)	86.8 (8.4)	88.3 (8.6)	**88.3 (7.3) ^p^**	**86.0 (10.5) ^p^**	85.8 (9.8)	88.8 (7.2)
[Median (IQR)]	87.0 (84.7–91.2)	87.2 (83.6–94.1)	89.3 (83.6–94.9)	**91.3 (85.7–92.8) ^p^**	**89.3 (84.5–92.2) ^p^**	89.3 (78.6–91.6)	90.6 (85.9–93.4)
Intermediate M-DC8+ (%) [Mean (SD)]	15.5 (7.7)	**17.9 (10.6) ^j^**	18.8 (13.0)	15.2 (6.5)	17.2 (11.2)	**13.4 (7.7) ^j^**	14.6 (6.6)
[Median (IQR)]	11.9 (9.1–23.6)	**15.7 (7.8–26.6) ^j^**	16.1 (9.3–26.1)	14.1 (11.1–21.1)	16.0 (7.1–25.5)	**13.4 (6.7–20.0) ^j^**	14.7 (8.3–19.1)
Intermediate CCR2+ (%) [Mean (SD)]	89.3 (2.9)	88.6 (2.1)	89.0 (5.5)	89.6 (1.6)	90.1 (3.4)	89.6 (4.5)	89.6 (4.3)
[Median (IQR)]	88.8 (87.8–91.2)	88.8 (87.2–90.6)	91.3 (86.5–92.4)	90.0 (88.3–91.1)	90.6 (87.7–92.9)	91.0 (87.9–92.2)	89.4 (88.9–93.2)
Non classical monocytes							
Non classical (CD16++CD14−) (%) [Mean (SD)]	11.0 (8.8)	11.4 (7.5)	12.1 (7.6)	12.9 (10.2)	13.0 (10.9)	12.3 (9.5)	14.3 (12.2)
[Median (IQR)]	9.3 (3.7–16.5)	10.7 (5.2–19.9)	10.2 (5.0–19.4)	7.1 (5.4–23.1)	8.3 (4.7–25.7)	12.2 (3.3–18.7)	8.6 (6.7–19.1)
Non classical CD163+ (%) [Mean (SD)]	31.2 (7.2) **^d^**	33.7 (9.1)	38.3 (15.9)	**35.3 (8.2) ^r^**	**35.7 (9.5) ^d^**	32.6 (6.3)	**30.9 (6.8) ^r^**
[Median (IQR)]	29.7 (26.3–38.0) **^d^**	35.0 (25.8–41.4)	31.6 (29.3–42.8)	**35.7 (30.0–40.0) ^r^**	**31.7 (30.2–46.4) ^d^**	31.8 (27.6–38.5)	**30.2 (25.6–35.7) ^r^**
Non classical CX3CR1+ (%) [Mean (SD)]	75.6 (11.5)	74.8 (14.0)	76.3 (14.8)	76.9 (12.2)	74.6 (15.5)	75.5 (12.4)	77.6 (14.9)
[Median (IQR)]	74.7 (71.6–84.0)	73.7 (67.2–85.9)	81.9 (61.1–87.6)	78.9 (65.5–86.6)	81.0 (57.4–83.8)	75.5 (63.6–87.9)	84.5 (60.3–88.7)
Non classical M-DC8+ (%) [Mean (SD)]	28.4 (10.3)	30.8 (15.3)	31.3 (18.6)	25.6 (7.5)	28.8 (10.8)	23.2 (7.0)	26.8 (10.5)
[Median (IQR)]	24.7 (21.5–39.8)	28.5 (17.8–37.7)	25.1 (20.5–34.4)	27.2 (19.1–32.5)	31.0 (17.9–37.6)	23.4 (17.6–26.8)	24.7 (18.7–37.5)
Non classical CCR2+ (%) [Mean (SD)]	**7.2 (3.3) ^e^**	8.1 (2.4)	8.1 (4.7)	7.2 (5.9)	7.9 (3.3)	**12.1 (8.2) ^e^**	7.9 (3.8)
[Median (IQR)]	**6.3 (4.5–10.5) ^e^**	7.8 (6.8–9.6)	8.3 (5.4–9.3)	5.0 (3.9–8.8)	6.8 (5.7–11.3)	**9.3 (6.7–18.1) ^e^**	7.9 (5.1–11.2)
Dendritic cells (DC)							
Plasmacytoid DC (CD123+CD11c−) (%) [Mean (SD)]	4.4 (2.1)	3.6 (1.1)	3.8 (1.3)	3.6 (1.5)	4.2 (1.1)	3.8 (1.7)	4.1 (1.1)
[Median (IQR)]	3.7 (2.7–5.5)	3.8 (2.5–4.4)	3.7 (3.1–5.1)	3.7 (2.4–4.4)	4.1 (3.2–5.0)	3.8 (2.5–4.7)	4.2 (3.3–4.9)
Myeloid DC (CD123−CD11c+) (%) [Mean (SD)]	**8.8 (3.2) ^b,e^**	11.5 (2.7)	**12.3 (4.0) ^b^**	10.3 (7.9)	**8.8 (3.9) ^s^**	**12.5 (4.8) ^e,s^**	12.6 (6.6)
[Median (IQR)]	**8.1 (7.0–10.4) ^b,e^**	11.2 (9.4–14.1)	**11.8 (9.9–13.7) ^b^**	7.8 (5.4–12.0)	**8.1 (5.7–10.5) ^s^**	**11.5 (9.4–15.6) ^e,s^**	13.0 (6.4–17.7)

Results are shown as the mean and standard deviation (SD) and as the median and interquartile range (IQR). Significant differences (*p* < 0.05) following the Wilcoxon matched-pairs signed-rank test are mentioned as follows: ^a^: week 0 vs. week 1; ^b^: week 0 vs. week 2; ^c^: week 0 vs. week 6; ^d^: week 0 vs. week 8; ^e^: week 0 vs. week 12; ^f^: week 0 vs. week 14; ^g^: week 1 vs. week 2; ^h^: week 1 vs. week 6; ^i^: week 1 vs. week 8; ^j^: week 1 vs. week 12; ^k^: week 1 vs. week 14; ^l^: week 2 vs. week 6; ^m^: week 2 vs. week 8; ^n^: week 2 vs. week 12; ^o^: week 2 vs. week 14; ^p^: week 6 vs. week 8; ^q^: week 6 vs. week 12; ^r^: week 6 vs. week 14; ^s^: week 8 vs. week 12; ^t^: week 8 vs. week 14; ^u^: week 12 vs. week 14. Significant values are presented in bold. ^$^: Two participants from CBD-only were excluded from analyses at week 6 (one participant) and weeks 8, 12, and 14 (two participants) because they were withdrawn at week 6 for safety concerns.

**Table 4:** Dynamic of HIV DNA and cell-associated RNA levels in CD4 T-cells isolated from blood and semen during cannabinoids treatment.

HIV DNA and RNA	Study Timeline						
	Week 0 Treatment Initiation *n* = 10	Week 1 *n* = 10	Week 2 *n* = 10	^$^ Week 6 *n* = 9	^$^ Week 8 *n* = 8	^$^ Week 12 End of Treatment *n* = 8	^$^ Week 14 Study Termination *n* = 8
Total HIV DNA (copies/10^6^ CD4) [Mean (SD)]	1016 (1081)	908.1 (817.3)	920.4 (816.5)	1053 (1121)	1292 (1310)	979.1 (1015)	1143 (1318)
[Median (IQR)]	708.1 (125.1–1679)	765.3 (239.3–1449)	819.8 (176.2–1529)	911.9 (141- 1886)	790.3 (165.6–2843)	629.9 (155.5–2156)	658.5 (152.3–2317)
LTR-gag cell-associated RNA (copies/10^6^ CD4) [Mean (SD)]	493.5 (557.3)	**593.9 (817.3) ^i^**	529.2 (630.3)	476.3 (479.6)	**1370 (1687) ^i,s^**	**572.9 (752.8) ^s^**	751.9 (858.3)
[Median (IQR)]	308.8 (4.9–915.8)	**224.3 (3.5–1088) ^i^**	271.2 (13.4–1081)	281.1 (21.9–938.8)	**142 (10.1–3151) ^i,s^**	**283.9 (2.1–1290) ^s^**	386.9 (26.0–1770)
RNA/DNA ratio [Mean (SD)]	0.47 (0.45)	0.56 (0.55)	0.43 (0.41)	0.43 (0.37)	**0.62 (0.62) ^s^**	**0.44 (0.53) ^s^**	1.85 (3.53)
[Median (IQR)]	0.3 (0.2–1.0)	0.46 (0.08–0.87)	0.37 (0.08–0.76)	0.33 (0.12–0.84)	**0.44 (0.05–1.26) ^s^**	**0.17 (0.01–1.07) ^s^**	0.50 (0.28–1.54)

Results are shown as the mean and standard deviation (SD) and as the median and interquartile range (IQR). Significant differences (*p* < 0.05) following the Wilcoxon matched-pairs signed-rank test are mentioned as follows: ^a^: week 0 vs. week 1; ^b^: week 0 vs. week 2; ^c^: week 0 vs. week 6; ^d^: week 0 vs. week 8; ^e^: week 0 vs. week 12; ^f^: week 0 vs. week 14; ^g^: week 1 vs. week 2; ^h^: week 1 vs. week 6; ^i^: week 1 vs. week 8; ^j^: week 1 vs. week 12; ^k^: week 1 vs. week 14; ^l^: week 2 vs. week 6; ^m^: week 2 vs. week 8; ^n^: week 2 vs. week 12; ^o^: week 2 vs. week 14; ^p^: week 6 vs. week 8; ^q^: week 6 vs. week 12; ^r^: week 6 vs. week 14; ^s^: week 8 vs. week 12; ^t^: week 8 vs. week 14; ^u^: week 12 vs. week 14. Significant values are presented in bold. ^$^: Two participants from CBD-only were excluded from analyses at week 6 (one participant) and weeks 8, 12, and 14 (two participants) because they were withdrawn at week 6 for safety concerns.

## Data Availability

Anonymized data may be available upon reasonable request of the author and the CTN.

## Supplementary Materials

The following supporting information can be downloaded at: https://www.mdpi.com/article/10.3390/cells12141811/s1, Table S1. List of antibodies used for ex vivo phenotyping of T-cell monocytes and dendritic cell subsets. Material S1. Detailed methodology for total HIV DNA and cell-associated and cell-free RNA quantification. Figure S1: Examples of gating on CD4 T-cell memory subsets (naïve: CD45RA+CD28+CCR7+, central memory: CD45RA−CD28+CCR7+, transitional memory: CD45RA−CD28+CCR7−, effector memory: CD45RA−CD28−CCR7−, and terminally differentiated: CD45RA+CD28−CCR7−) and markers involved in CD4 T-cell function. Figure S2: Examples of gating on (a) CD4 helper T-cell subsets (Th17: CD45RA−CCR4+CCR6+ CXCR3−, Th1-Th17: CD45RA−CCR4−CCR6+CXCR3+, Th2: CD45RA−CCR4+CCR6−CXCR3−, Th1: CD45RA−CCR4−CCR6−CXCR3+) and (b) regulatory T-cells (CD25hi CD127lo FoxP3+). Figure S3: Examples of gating on markers involved in CD8 T-cell function. (c) Examples of gating on CD8 T-cell memory subsets (naïve: CD45RA+CD28+CCR7+, central memory: CD45RA−CD28+CCR7+, transitional memory: CD45RA−CD28+CCR7−, effector memory: CD45RA−CD28−CCR7−, and terminally differentiated: CD45RA+CD28−CCR7−) and regulatory CD8 T-cells (FoxP3+). Figure S4: (Examples of gating on classical (CD14++CD16−), intermediates (CD14+CD16+) and non-classical (CD14−CD16++) monocytes and myeloid (HLA-DR+CD123−CD11c+) and plasmacytoid (HLA-DR+CD123+CD11c−) dendritic cells. Figure S5: Examples of gating on markers of cell migration and function in (a) classical, (b) intermediate, and (c) non-classical monocytes.
