# Supplementary material for: Effects of Oral Cannabinoids on Systemic Inflammation and Viral Reservoir Markers in People with HIV on Antiretroviral Therapy: Results of the CTN PT028 Pilot Clinical Trial
Source: Cells. 2023 Jul 8;12(14):1811. doi: 10.3390/cells12141811 (PMC10378564; doi:10.3390/cells12141811)
Supplement: Supplementary file 1 [file cells-12-01811-s001.zip › cells-2394838-supplementary.pdf]

Table S1. List of antibodies used for *ex vivo* phenotyping of T-cell monocytes and dendritic cells subsets.

***Ex vivo* T-cell phenotyping**			
**EXTRACELLULAR**			
**mAb**	**Clone**	**Cat. No.**	**Company**
BV786 Mouse Anti-Human CD3	UCHT1	565491	BD Horizon
APC Mouse Anti-Human CD4	RPA-T4	555349	BD Pharmigen
FITC Mouse Anti-Human CD4	RPA-T4	555346	BD Pharmigen
APC-R700 Mouse Anti-Human CD8	RPA-T8	565165	BD Horizon
BV421 Mouse Anti-Human CD194 (CCR4)	1G1	562579	BD Horizon
BB515 Mouse Anti-Human CD196 (CCR6)	11A9	564479	BD Horizon
PE-Cy™5 Mouse Anti-Human CD183 (CXCR3)	1C6/CXCR3	551128	BD Pharmigen
APC-H7 Mouse Anti-Human CD45RA	HI100	561212	BD Pharmigen
PE Mouse Anti-Human CD25	M-A251	555432	BD Pharmigen
PE-Cy™7 Mouse Anti-Human CD127	HIL-7R-M21	560822	BD Pharmigen
BV711 Mouse Anti-Human CD39	TU66	563680	BD Horizon
PerCP-Cy™5.5 Mouse anti-Human CD197	150503	561144	BD Pharmigen
APC Mouse Anti-Human CD28	28.2	559770	BD Pharmigen
BV605 Mouse Anti-Human HLA-DR	G46-6	562845	BD Horizon
PE-Cy™7 Mouse Anti-Human CD38	HIT2	560677	BD Pharmigen
BV421 Mouse Anti-Human CD57	NK-1	563896	BD Horizon
BioLegend PE anti-human CD279 (PD-1)	EH12.2H7	329906	Biolegend
BV605 Mouse Anti-Human CD73	AD2	563199	BD Horizon
**INTRACELLULAR**			
PE-CF594 Mouse Anti-Human FoxP3	236A/E7	563955	BD Horizon
BV711 Mouse Anti-Ki-67	B56	563755	BD Horizon
PE-CF594 Mouse Anti-Human CD152	BNI3	562742	BD Horizon
***Ex vivo* monocytes and dendritic cells phenotyping**			
**EXTRACELLULAR**			
BV605 Mouse Anti-Human HLA-DR	G46-6	562845	BD Horizon
BV786 Mouse Anti-Human CD14	M5E2	563699	BD Horizon
Alexa Fluor® 700 Mouse Anti-Human CD16	3G8	302026	BioLegend
BV711 Mouse Anti-Human CD11c	B-ly6	563130	BD Horizon
PE-CF594 Mouse Anti-Human CD123	7G3	562391	BD Horizon
APC-Cy7 Mouse Anti-Human CD163	GHI/61	333622	BioLegend
PE-Cy7 Mouse Anti-Human CD192 (CCR2)	K036C2	357212	BioLegend
BV650 Rat Anti-Human CX3CR1	2A9-41	341626	BioLegend
FITC Mouse Anti-Human Slan (M-DC8)	DD-1	130-117-371	Miltenyi

Material S1. Detailed methodology for total HIV DNA and cell-associated and cell free RNA quantification

Quantification of total HIV DNA

The frequency of cells harbouring total HIV DNA (copies per million cells) was determined using an ultrasensitive PCR-based assay (sensitivity of 1 copy/PCR reaction) as previously described (Vandergeteen et al. JVI, 2014), but with minor modifications to the original protocol. Genomic DNA from PBMCs, and semen cell pellets was extracted using the QIAamp DNA mini kit (Qiagen), and Total HIV DNA was amplified from extracted total genomic DNA by nested real time PCR with primers and probes specific for the LTR/gag region. A pre-amplification of total HIV DNA was first carried out in a 50-µl reaction mixture containing the following HIV primers: ULF1: 5'- ATG CCA CGT AAG CGA AAC TCT GGG TCT CTC TDG TTA GAC-3'; UR1: 5’- CCA TCT CTC TCC TTC TAG C -3'. Primers specific for the human CD3 gene (LambdaT: 5'-ATG CCA CGT AAG CGA AAC T -3’; UR2: 5'- CTG AGG GAT CTC TAG TTA CC-3') were used to quantify the exact number of genomes present in the reaction tube (Vandergeteen et al. JVI, 2014). Then, a second round of PCR was carried out in a final volume of 20 µl containing 6.4 µl of a 1/10 dilution of the first PCR products. Primers sets for CD3 and HIV, and the UHIV TaqMan probe 100nM (UHIV FamZen: 5'-/56-FAM/CA CTC AAG G/ZEN/C AAG CTT TAT TGA GGC /3IABkFQ/-3') were added to the master mix. The real-time PCR assay was performed on a Rotor-Gene Q instrument (Qiagen) with the PerfeCta qPCR ToughMix (Quanta Biosciences) following the manufacturer’s instructions. ACH-2 cells, which carry a single copy of the integrated HIV genome, were used to generate a standard curve. Serial 10-fold dilutions of ACH-2 cells ranging from 3 x 10^5 to 3 cells were amplified together with experimental samples and were used as standards for both HIV and CD3 gene quantifications.

Quantification of cell associated HIV RNA

Cellular and cell-free RNA were extracted from blood and semen cell pellets and from semen supernatant, respectively using the QIAamp RNA mini kit (Qiagen) and HIV RNA (LTR /gag) was quantified using a similar approach and similar primers and probes than for total HIV DNA. Briefly, genomic DNA contaminants were removed from cellular extracted RNA using DNase I (Invitrogen), following the manufacturer’s instructions. Reverse transcription and pre-amplification were performed in a single step reaction using the superscript III one-step RT-PCR system (Invitrogen). Primers specific for GUSB RNA were used as an endogenous control to normalize on the cellular input present in the reaction tube, using the following primers and probes: (Forward 1: 5’-ACC TAG AAT CTG CTG GCT ACT A-3’; Reverse 1: 5’- GTT CAA ACA GAT CAC ATC CAC ATA C-3’; Forward 2: 5'-TGC TGG CTA CTA CTT GAA GAT G-3’; Reverse 2: 5'- CCT TGT CTG CTG CAT AGT TAG A-3'; Probe: 5'-/5HEX/TCGCTCACA/ZEN/CCAAATCCTTGGACC/3IABkFQ/-3'). LTR-Gag transcripts produced in vitro were used as a standard for the quantification of HIV RNA. Serial 10-fold dilutions ranging from 3 x 10^5 to 3 copies of HIV RNA were amplified together with experimental samples.

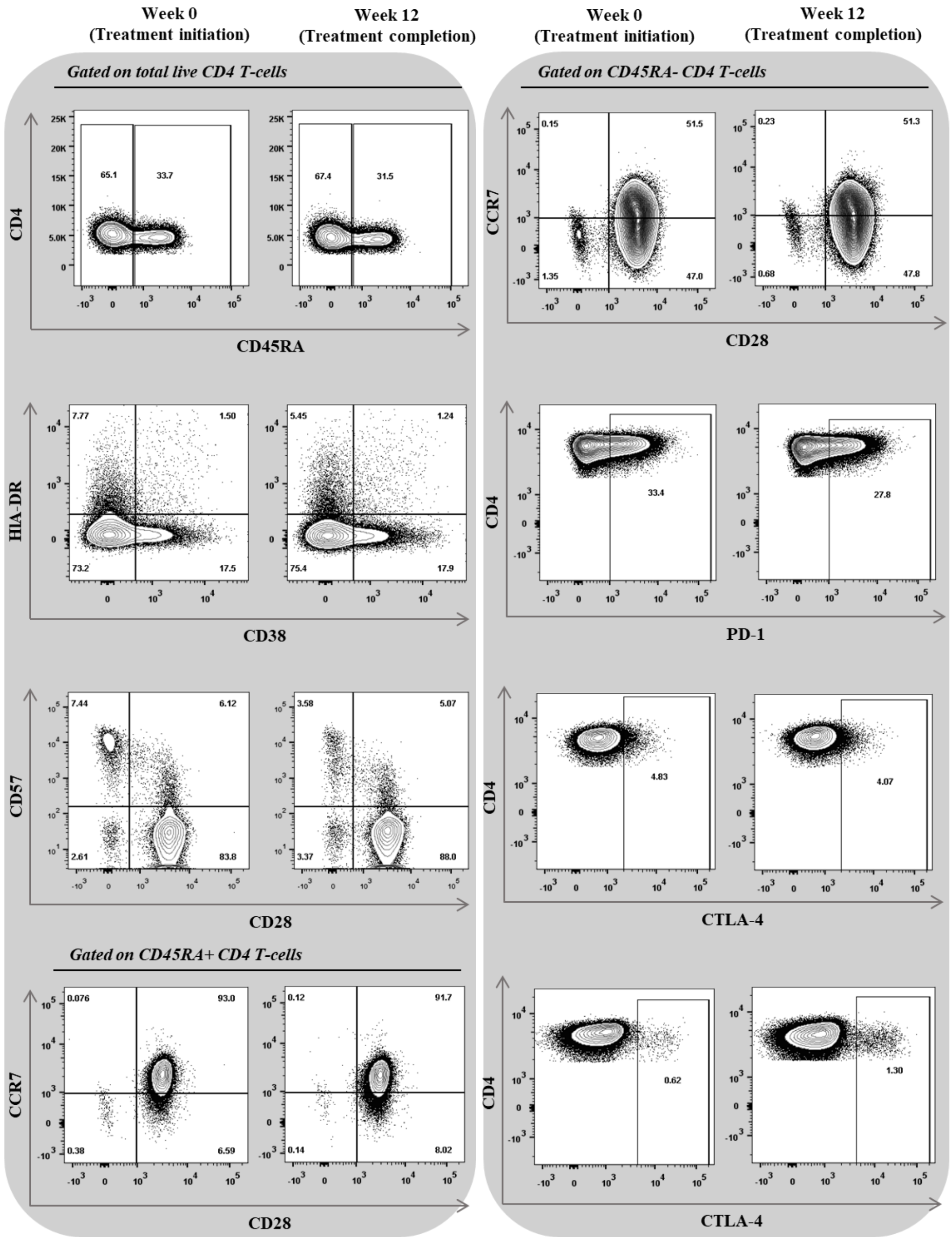

Figure S1: Examples of gating on CD4 T-cell memory subsets (Naïve: CD45RA+CD28+CCR7+; Central memory: CD45RA-CD28+CCR7+; Transitional memory: CD45RA-CD28+CCR7-; Effector memory: CD45RA-CD28-CCR7-; Terminally differentiated: CD45RA+CD28-CCR7-) and markers involved in CD4 T-cell function.

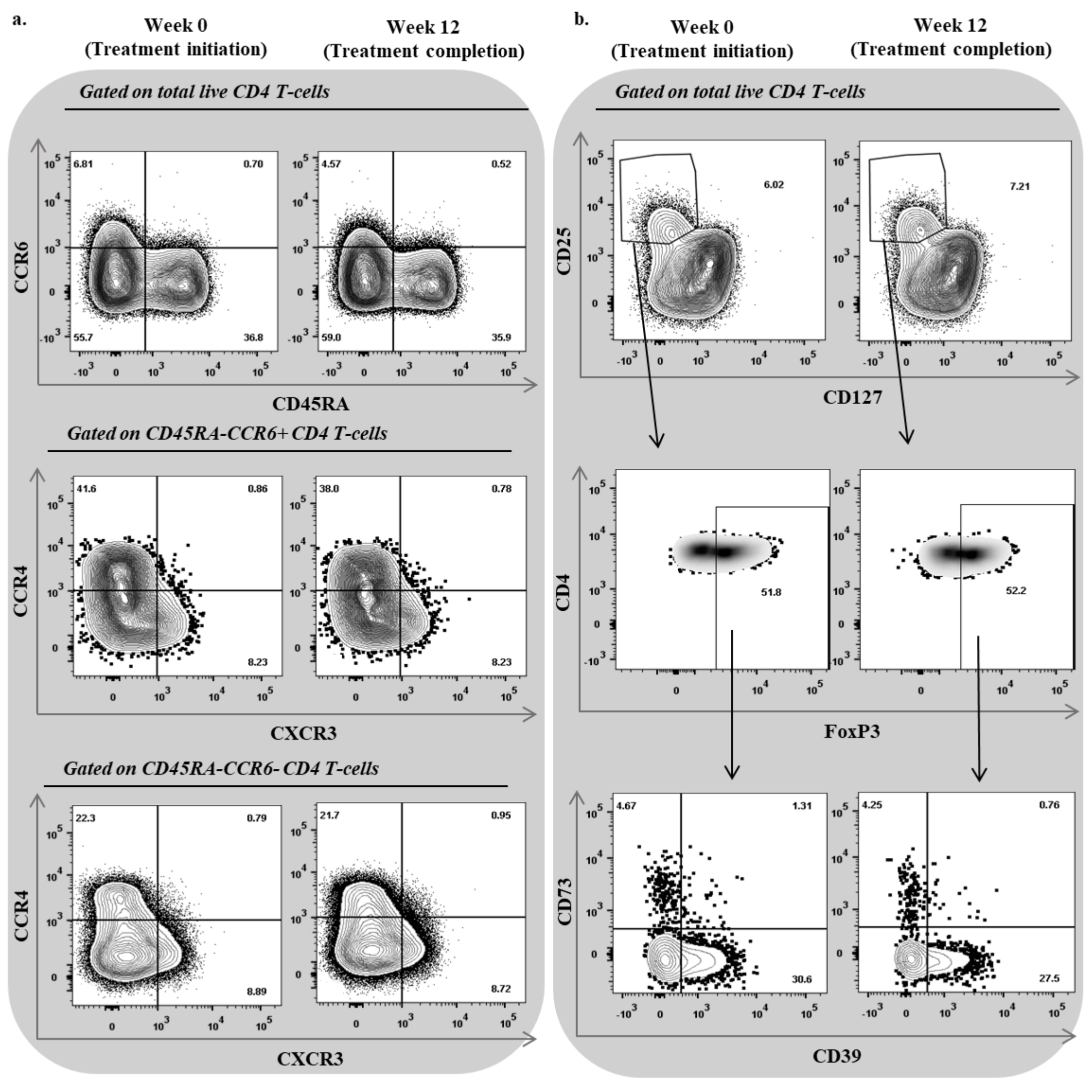

Figure S2: Examples of gating on **(a)** CD4 helper T-cell subsets (Th17: CD45RA-CCR4+CCR6+ CXCR3-; Th1-Th17: CD45RA-CCR4-CCR6+CXCR3+; Th2: CD45RA-CCR4+CCR6- CXCR3-; Th1: CD45RA-CCR4-CCR6- CXCR3+) and **(b)** regulatory T-cells (CD25hi CD127lo FoxP3+).

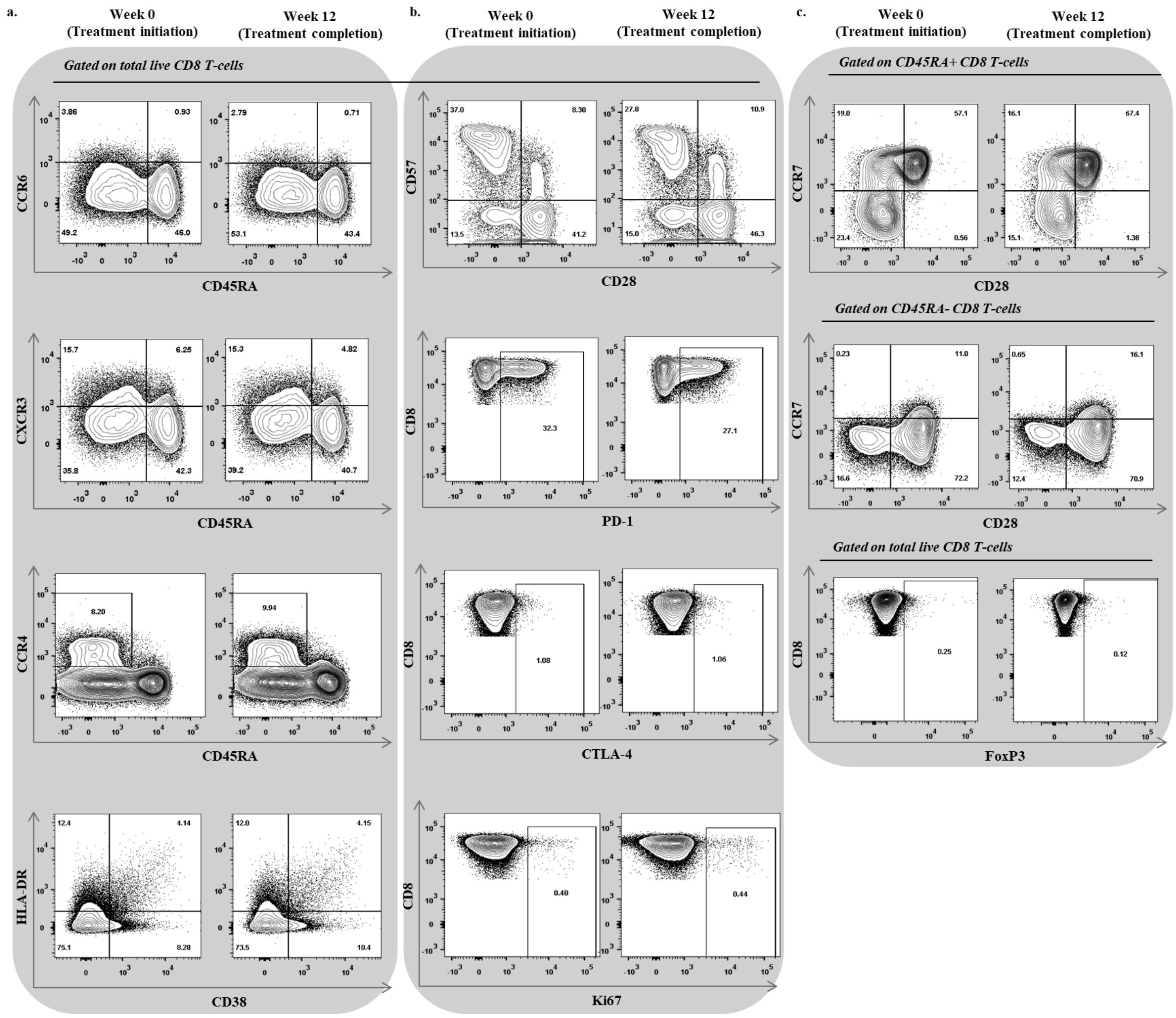

Figure S3: (a,b) Examples of gating on markers involved in CD8 T-cell function. **(c)** Examples of gating on CD8 T-cell memory subsets (Naïve: CD45RA+CD28+CCR7+; Central memory: CD45RA-CD28+CCR7+; Transitional memory: CD45RA-CD28+CCR7-; Effector memory: CD45RA-CD28-CCR7-; Terminally differentiated: CD45RA+CD28-CCR7-) and regulatory CD8 T-cells (FoxP3+).

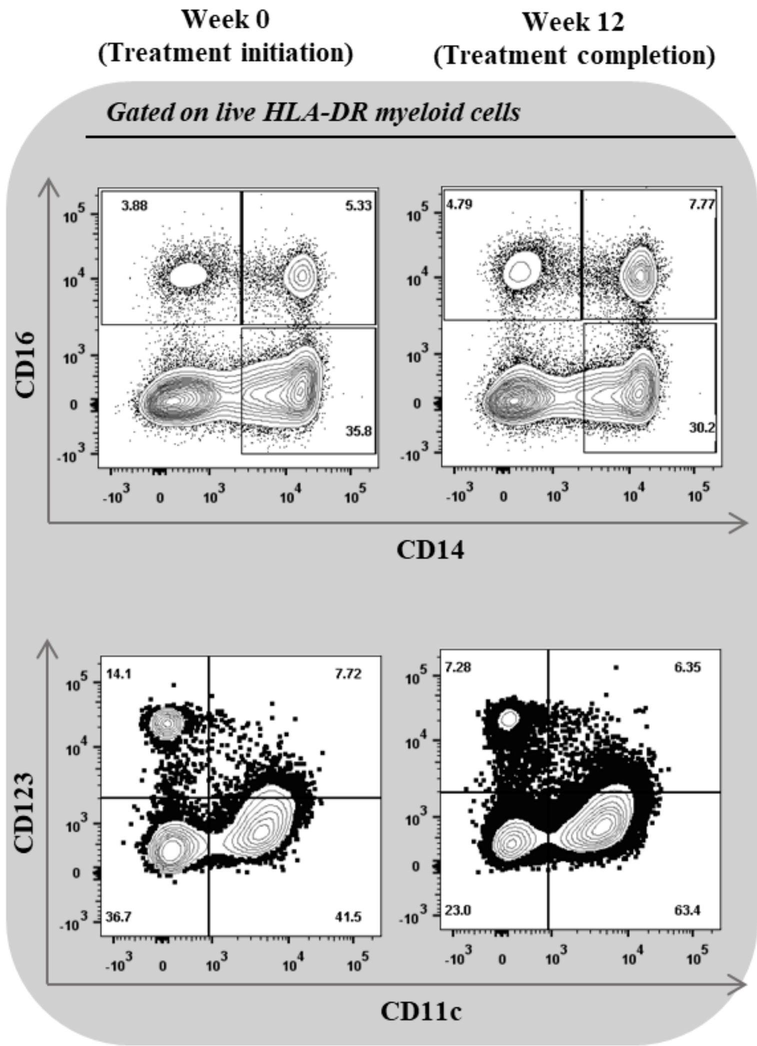

Figure S4: (Examples of gating on classical (CD14++CD16-), intermediates (CD14+CD16+) and non-classical (CD14-CD16++) monocytes and myeloid (HLA-DR+CD123-CD11c+) and plasmacytoid (HLA-DR+CD123+CD11c-) dendritic cells.

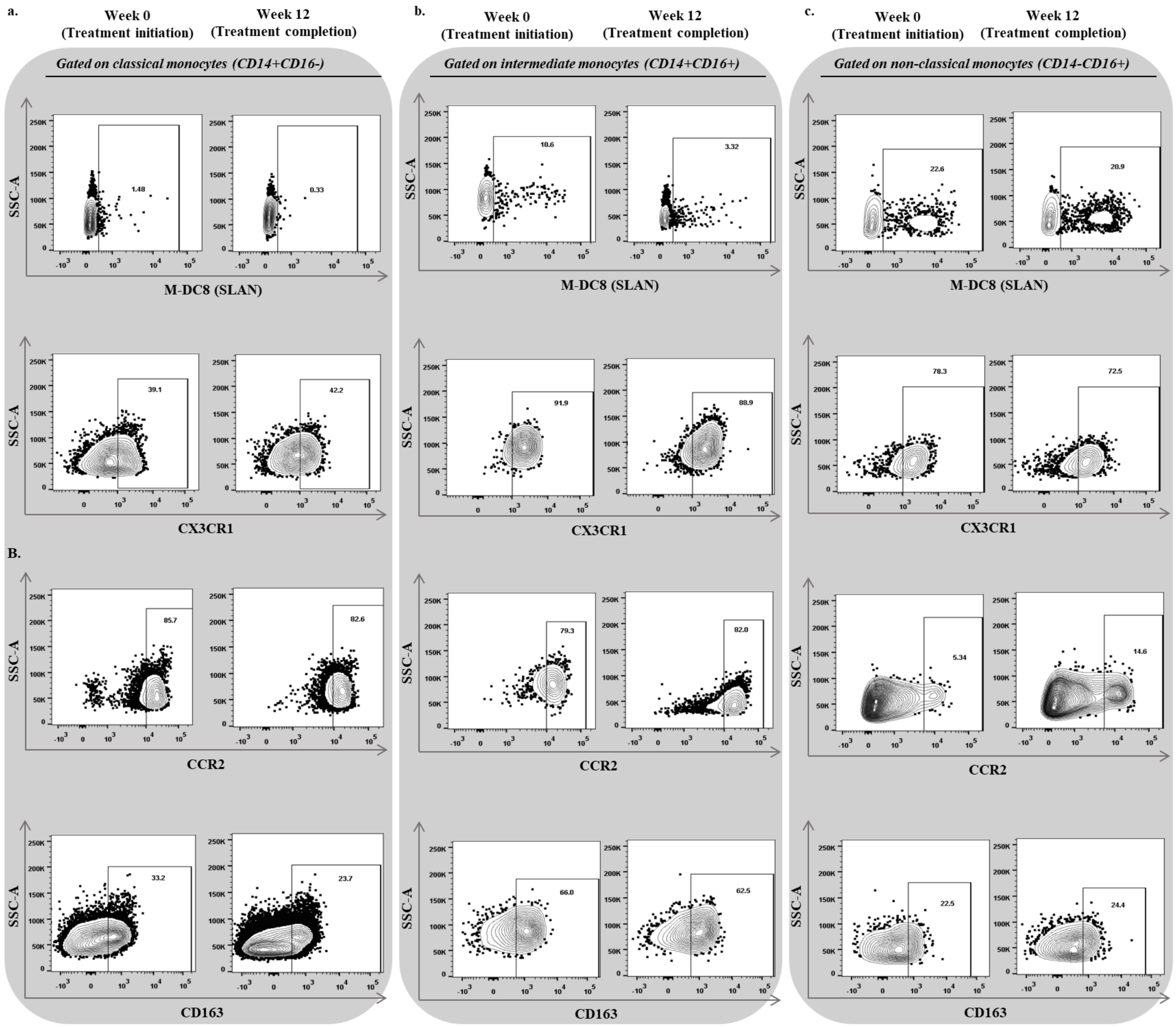

Figure S5: Examples of gating on markers of cell migration and function in **(a)** classical, **(b)** intermediate, and **(c)** non-classical monocytes.
